# Electroluminescent Warm White Light‐Emitting Diodes Based on Passivation Enabled Bright Red Bandgap Emission Carbon Quantum Dots

**DOI:** 10.1002/advs.201900397

**Published:** 2019-04-30

**Authors:** Haoran Jia, Zhibin Wang, Ting Yuan, Fanglong Yuan, Xiaohong Li, Yunchao Li, Zhan'ao Tan, Louzhen Fan, Shihe Yang

**Affiliations:** ^1^ College of Chemistry Beijing Normal University Beijing 100875 China; ^2^ State Key Laboratory of Alternate Electrical Power System with Renewable Energy Sources North China Electric Power University Beijing 102206 China; ^3^ Guangdong Key Lab of Nano‐Micro Material Research School of Chemical Biology and Biotechnology Shenzhen Graduate School Peking University Shenzhen 518055 China

**Keywords:** carbon quantum dots, electroluminescence, high quantum yield, red fluorescence, warm white light‐emitting diodes

## Abstract

The development of efficient red bandgap emission carbon quantum dots (CQDs) for realizing high‐performance electroluminescent warm white light‐emitting diodes (warm‐WLEDs) represents a grand challenge. Here, the synthesis of three red‐emissive electron‐donating group passivated CQDs (R‐EGP‐CQDs): R‐EGP‐CQDs‐NMe_2_, ‐NEt_2_, and ‐NPr_2_ is reported. The R‐EGP‐CQDs, well soluble in common organic solvents, display bright red bandgap emission at 637, 642, and 645 nm, respectively, reaching the highest photoluminescence quantum yield (QY) up to 86.0% in ethanol. Theoretical investigations reveal that the red bandgap emission originates from the rigid π‐conjugated skeleton structure, and the ‐NMe_2_, ‐NEt_2_, and ‐NPr_2_ passivation plays a key role in inducing charge transfer excited state in the π‐conjugated structure to afford the high QY. Solution‐processed electroluminescent warm‐WLEDs based on the R‐EGP‐CQDs‐NMe_2_, ‐NEt_2_, and ‐NPr_2_ display voltage‐stable warm white spectra with a maximum luminance of 5248–5909 cd m^−2^ and a current efficiency of 3.65–3.85 cd A^−1^. The warm‐WLEDs also show good long‐term operational stability (*L*/*L*_0_ > 80% after 50 h operation, *L*_0_: 1000 cd m^−2^). The electron‐donating group passivation strategy opens a new avenue to realizing efficient red bandgap emission CQDs and developing high‐performance electroluminescent warm‐WLEDs.

## 1 Introduction

White light‐emitting diodes (WLEDs) have become a strong contender as the future solid‐state lighting sources owing to their advantages of high luminous efficiency, high luminance, and energy conservation.^1^, ^2^ WLEDs are generally classified into two categories, i.e., phosphor‐converted WLEDs (pc‐WLEDs) and electroluminescent WLEDs.^3^ pc‐WLEDs, where a ultraviolet‐ (UV‐) or blue‐LED optically pumping phosphors toward point white‐light sources,^4^, ^5^ are susceptible to energy losses due to reabsorption, scattering, thermal quenching, and photobleaching.^6^ By contrast, electroluminescent WLEDs rely on direct charge carrier injection into the light‐emitting layer without energy losses, thus permitting full use of the easy solution‐processing to achieve area white‐light sources with higher efficiency.^7^, ^8^ On the other hand, warm white light with low correlated color temperature (CCT < 4000 K) is strongly desired for being physiological compatible and readily creating a healthy and comfortable atmosphere at night.^9^, ^10^ The crucial issue involved with achieving warm white EL is the rational design of active‐emitting layer (EML), where a red electroluminescent material (REM) is undoubtedly essential in complementing the red light component and lowering the CCT of electroluminescent WLEDs.^11^, ^12^ To date, with steady efforts devoted to the material chemistry of Cd^2+^‐based colloidal semiconductor quantum dots (Cd^2+^‐QDs), red emissive Cd^2+^‐QDs with high photoluminescence quantum yield (QY),^13^ narrow emission and good organic solubility are now available for fabricating QDs‐based electroluminescent warm‐WLEDs.^14^, ^15^ Nevertheless, limited by the intrinsic narrow emission bandwidths of Cd^2+^‐QDs, the design principle of aforementioned devices only allows to form the randomly mixed emissive layer with three or more colored QDs for covering the visible spectrum, which inevitably leads to a severe problem of electroluminescence spectral fluctuation against voltage variation because of uncontrollable carriers flowing processes among QDs with diverse band structures.^14^, ^15^ Moreover, the potential toxic and detrimental effects on humans and environment derived from Cd^2+^‐QDs are other inherent problems.^16^ It is thus of paramount importance to develop REM with high QY, favorable organic solubility, broad emission bandwidths, and nontoxicity for electroluminescent warm‐WLEDs.

Carbon quantum dots (CQDs) with intrinsic bandgap emission have aroused intense interests in electroluminescent LEDs owing to their tunable photoluminescence (PL), high stability, low cost, and nontoxicity, which is thought to be an ideal alternative to Cd^2+^‐QDs.^17^, ^18^, ^19^, ^20^, ^21^, ^22^, ^23^ Monochrome LEDs from blue to red have been developed in our lab based on the high quality CQDs.^18^ Straight after that, red emissive CQDs with 53% QY (λ_em_ = 628 nm in ethanol) is synthesized and applied to high color rendering and stable warm pc‐WLEDs.^9^ Nevertheless, red emissive CQDs with even higher QY as well as good organic solubility have hardly been reported, but greatly needed for realizing high performance solution‐processed warm‐WLEDs.^24^, ^25^, ^26^, ^27^, ^28^, ^29^ Lin and co‐workers reported efficient red emissive CQDs (λ_em_ = 604 nm in ethanol, 26.1% QY) derived from solvothermal treatment of p‐phenylenediamine (p‐PD) precursor.^24^ Herein we use *N*,*N*‐dimethyl‐, *N*,*N*‐diethyl‐, and *N*,*N*‐dipropyl‐p‐PD as precursors to synthesize red‐emissive electron‐donating group passivated CQDs (R‐EGP‐CQDs), R‐EGP‐CQDs‐NMe_2_, ‐NEt_2_, and ‐NPr_2_. The R‐EGP‐CQDs display bright red bandgap emission at 637, 642, and 645 nm, respectively, with the highest QY up to 86.0% in ethanol. They also exhibit good solubility in common organic solvents. Benefitting from the merits of as‐prepared R‐EGP‐CQDs, we realized the high performance solution‐processed electroluminescent warm‐WLEDs. The warm‐WLEDs display voltage‐stable warm white color and high‐performance with maximum luminance (*L*_max_) of 5248–5909 cd m^−2^ and maximum current efficiency (η_c,max_) of 3.65–3.85 cd A^−1^. Moreover, they also show good long‐term operation stability (*L*/*L*_0_ > 80% after 50 h operation, *L*_0_: 1000 cd m^−2^).

## 2 Results and Discussion

**Figure 1:**
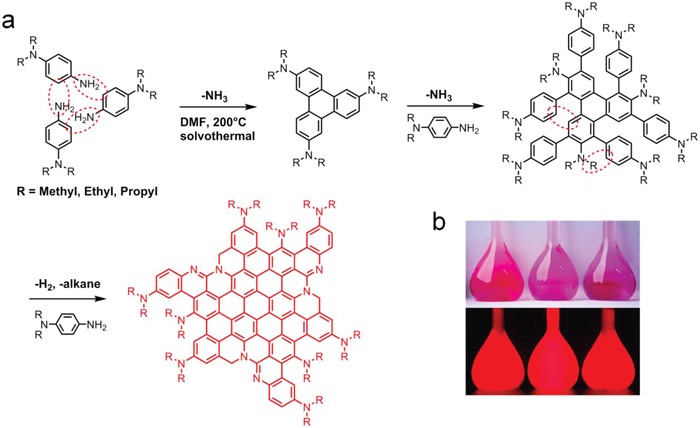
a) Synthesis of R‐EGP‐CQDs‐NMe_2_, ‐NEt_2_, and ‐NPr_2_ by solvothermal treatment of *N*,*N*‐dimethyl‐, *N*,*N*‐diethyl‐, and *N*,*N*‐dipropyl‐p‐PD, respectively. b) Photographs of R‐EGP‐CQDs‐NMe_2_ (left), ‐NEt_2_ (middle), and ‐NPr_2_ (right) dilute ethanol solution (0.1 mg mL^−1^) under daylight (top) and 365 nm UV light (bottom).

Synthesis of R‐EGP‐CQDs‐NMe_2_, ‐NEt_2_, and ‐NPr_2_ were carried out based on the reported method,^24^ involving the solvothermal treatment of *N*,*N*‐dimethyl‐, *N*,*N*‐diethyl‐, and *N*,*N*‐dipropyl‐p‐PD as precursors in dimethyl formamide (DMF) (5 mg mL^−1^) at 200 °C for 12 h, respectively (**Figure** **1**a). The crude products were purified via silica column chromatography to give a considerable product yield of 20%. As shown in Figure 1b, three R‐EGP‐CQDs solutions display red appearance under daylight (Figure 1b top) and bright red PL under UV light (Figure 1b bottom).

**Figure 2:**
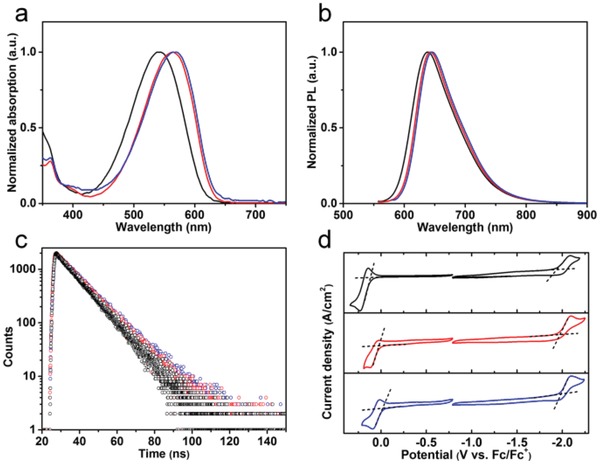
a) Normalized UV–vis absorption, b) PL emission spectra, c) TRPL spectra, and d) CV curves of R‐EGP‐CQDs‐NMe_2_ (black), ‐NEt_2_ (red), and ‐NPr_2_ (blue).

R‐EGP‐CQDs‐NMe_2_, ‐NEt_2_, and ‐NPr_2_ (0.1 mg mL^−1^ in ethanol) exhibit typical excitonic absorption band centered at 540, 563, and 568 nm from the ultraviolet–visible (UV–vis) absorption spectra, respectively (**Figure** **2**a). Their PL emission spectra display excitonic emission peaks centered at 637, 642, and 645 nm, respectively (Figure 2b). The PL excitation spectra fit well with the corresponding UV–vis absorption spectra (Figure S1, Supporting Information), and are irrespective of excitation wavelength in a wide range (Figure S2, Supporting Information), which demonstrate that their emissions stem from intrinsic band‐edge transition.^9^, ^19^ Time‐resolved photoluminescence (TRPL) analysis reveals the PL emissions are characterized by monoexponential decay, which is on nanosecond timescale (10.8, 11.4, and 11.8 ns for R‐EGP‐CQDs‐NMe_2_, ‐NEt_2_, and ‐NPr_2_, respectively), implying the singlet state nature of PL emission and only one radiative transition channel accounting for the emission (Figure 2c).^19^, ^30^ The absolute QYs of R‐EGP‐CQDs‐NMe_2_, ‐NEt_2_, and ‐NPr_2_ are determined to be 77.9%, 85.2%, and 86.0% in ethanol with the corresponding excitation wavelength being 540, 560, and 560 nm, respectively. To the best of our knowledge, the 86.0% reported here are the highest QY for red emissive CQDs reported to date (Table S1, Supporting Information).^9^, ^18^, ^19^, ^24^, ^25^, ^26^, ^27^, ^28^, ^29^ The optical bandgap (*E*_g,opt_) extracted from the absorption edge of R‐EGP‐CQDs‐NMe_2_, ‐NEt_2_, and ‐NPr_2_ gradually dereceases from 2.10 to 2.06 and 2.05 eV (Figure S3, Supporting Information). This is further confirmed by cyclic voltammegrams (CVs), which are measured in acetonitrile containing 0.1 m (Bu)_4_NPF_6_ as the supporting electrolyte (Figure 2d; Table S2, Supporting Information). The highest occupied molecular orbital (HOMO) and lowest unoccupied molecular orbital (LUMO) levels are calculated from the onset potentials of oxidation (*E*_Ox_^onset^) and reduction (*E*_Red_^onset^), respectively.^31^ For R‐EGP‐CQDs‐NMe_2_, ‐NEt_2_, and ‐NPr_2_, *E*_Ox_^onset^ decrease from 0.12 to 0.02 and −0.02 V, while *E*_Red_^onset^ almost remain constant (slightly decreased) from −1.96 to −1.98 and −1.99 V, thus giving a gradually decreased electrochemical bandgap (*E*_g,CV_) of 2.08, 2.00, and 1.97 eV. The HOMO levels for R‐EGP‐CQDs‐NMe_2_, ‐NEt_2_, and ‐NPr_2_ are determined to be −4.92, −4.82, and −4.78 eV, respectively, while the LUMO levels are located at −2.84, −2.82, and −2.81 eV, respectively.

**Figure 3:**
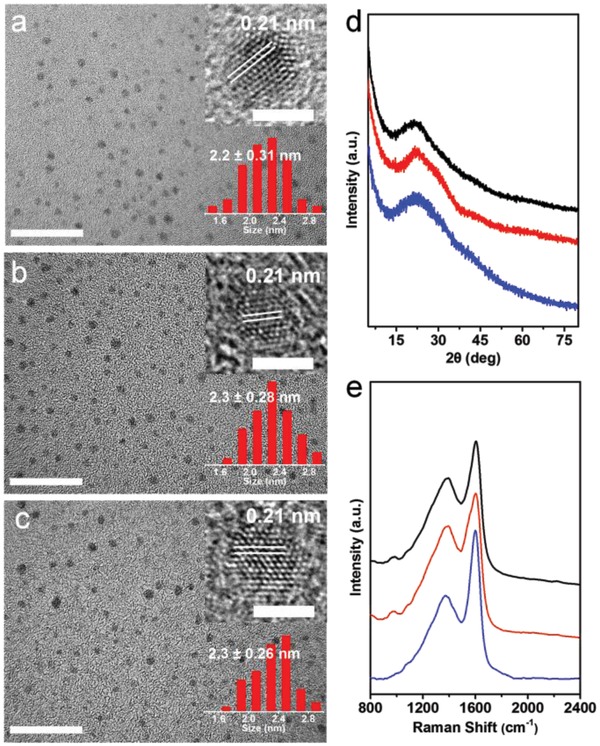
TEM images (scale bar: 20 nm), corresponding HRTEM photographs (top right insets, scale bar: 2 nm) and statistical size distributions (low right insets) of a) R‐EGP‐CQDs‐NMe_2_, b) ‐NEt_2_, and c) ‐NPr_2_. d) XRD patterns and e) Raman spectra of R‐EGP‐CQDs‐NMe_2_ (black), ‐NEt_2_ (red), and ‐NPr_2_ (blue).

The transmission electron microscopy (TEM) images reveal that R‐EGP‐CQDs‐NMe_2_, ‐NEt_2_, and ‐NPr_2_ are monodispersed nanodots with average lateral size of 2.2 ± 0.31, 2.3 ± 0.28 to 2.3 ± 0.26 nm (**Figure** **3**a–c). The high‐resolution TEM (HRTEM) images demonstrate the high crystallinity with a lattice spacing of 0.21 nm corresponding to (100) lattice spacing of graphene along the [001] direction (top right insets in Figure 3a–c).^32^ Their atomic force microscopy (AFM) images reveal the topographic heights are mostly in the range from 1 to 2 nm corresponding to 3–6 graphene layers (Figure S4, Supporting Information).^26^, ^33^ A wide reflection peak centered at around 26° is observed in X‐ray powder diffraction (XRD) patterns (Figure 3d), showing *d* spacing of 0.34 nm, which is assigned to the graphite lattice spacing of (002) facet.^19^, ^26^ In Raman spectra (Figure 3e), the ordered G bands centered at around 1600 cm^−1^ are stronger than the disordered D band centered at 1380 cm^−1^ with a large *I*_D_/*I*_G_ ratio of 1.67, 1.72, and 1.74, for R‐EGP‐CQDs‐NMe_2_, ‐NEt_2_, and ‐NPr_2_, respectively.

**Figure 4:**
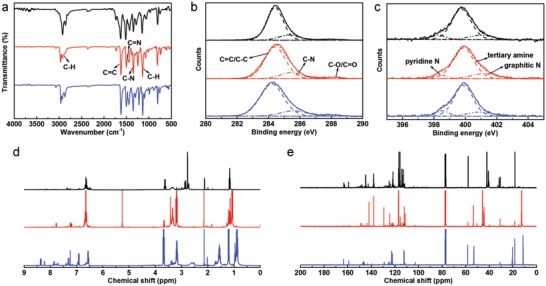
a) FTIR, b) high resolution C_1s_ XPS, c) high resolution N_1s_ XPS, d) ^1^H NMR, and e) ^13^C NMR spectra of R‐EGP‐CQDs‐NMe_2_ (black), ‐NEt_2_ (red), and ‐NPr_2_ (blue).

In the Fourier transform infrared (FTIR) spectra of R‐EGP‐CQDs‐NMe_2_, ‐NEt_2_, and ‐NPr_2_ (**Figure** **4**a), the formation of π‐conjugated structure is verified with the observation of aromatic C=C stretching vibrations (1620–1631 cm^−1^) and aromatic C—H in‐plane bending vibrations (1138–1149 cm^−1^).^32^ Two characteristic N—H stretching vibrations (3442, 3360 cm^−1^) of primary amine (—NH_2_) vanish in all three CQDs. On the other hand, Peaks observed at 3000–2850 and 1221–1276 cm^−1^ are attributed to aliphatic C—H and aliphatic C—N stretching vibrations, respectively.^18^ These evidences qualitatively suggest that the tertiary amine groups (—NR_2_, where R is Me, Et, and Pr, respectively) from precursors retains in R‐EGP‐CQDs‐NMe_2_, ‐NEt_2_, and ‐NPr_2_, while the —NH_2_ group from precursor might largely undergoes deamination during solvothermal treatment. With the observation of aromatic C—N stretching vibrations at 1343–1349 cm^−1^, it confirms that —NR_2_ is directly linked to the π‐conjugated structure and serves as so‐called passivation group.^25^ Meanwhile peaks observed at 1502–1513 cm^−1^ are attributed to C=N stretching vibrations.^30^ As shown in the X‐ray photoelectron spectroscopy (XPS) survey spectra (Figure S5, Supporting Information), the elemental compositions for the three CQDs are C (284.8–284.9 eV), N (399.8–400.0 eV), and O (531.1–531.4 eV). We also found a higher atomic ratio of C to N for R‐EGP‐CQDs‐NMe_2_, ‐NEt_2_, and ‐NPr_2_ (7.5:1, 9.3:1, and 11.2:1) than that for corresponding precursor (4:1, 5:1, and 6:1) (Table S3, Supporting Information), suggesting the occurrence of deamination during solvothermal treatment with respect to the —NH_2_ from precursors. In the high‐resolution C1s spectra, signals of C=C (284.2–284.5 eV), C—N (285.4–285.5 eV), and C=O (287.9–288.0 eV) are observed (Figure 4b; Table S4, Supporting Information).^18^ The high‐resolution N1s spectra (Figure 4c; Table S4, Supporting Information) can be fitted with three sublevels, and the main one (relative content of 77.5–78.1%) with peak at 399.9 eV is assigned to —NR_2_,^34^ confirming the FTIR results. The other two with peaks at 398.4–398.5 eV (relative content of 6.3–9.2%) and 400.5–410.0 eV (relative content of 13.3–15.6%) are assigned to pyridine N and graphitic N, respectively,^30^, ^35^ demonstrating the presence of N doping. Shown in Figure 4d,e are the ^1^H and ^13^C‐nuclear magnetic resonance (NMR) spectra of R‐EGP‐CQDs‐NMe_2_, ‐NEt_2_, and ‐NPr_2_ in deuterium dichloromethane. In the downfield of ^1^H and ^13^C‐NMR spectra, peaks appearing in the range of 6.5–8 and 110–150 ppm are classified as signals of aromatic hydrogen and carbon, respectively, confirming the presence of π‐conjugated structure.^19^ In the highfield of ^1^H and ^13^C‐NMR spectra, peaks appearing in the range of 1–4 ppm and <60 ppm are assigned to aliphatic hydrogen and carbon, respectively.^36^, ^37^ In addition, according to the quantitative analysis results based on the integral processing of ^1^H‐NMR spectra (Figures S6–S8, Supporting Information), different kinds of protons can be clearly distinguished from its chemical shift and integral intensity.^38^ The difference in the type of aliphatic signals for the three CQDs should arise from the corresponding precursors. Taken together, it is evident that R‐EGP‐CQDs‐NMe_2_, ‐NEt_2_, and ‐NPr_2_ are composed of large π‐conjugated structure which is highly crystalline, N doped and passivated by amounts of strong electron‐donating groups (EDGs), i.e., ‐NR_2_. According to the above structural analysis, we propose a possible reaction mechanism toward R‐EGP‐CQDs‐NMe_2_, ‐NEt_2_, and ‐NPr_2_ (Figure 1a). During the high temperature solvothermal treatment, precursor molecules react with each other through the deamination between —NH_2_ and beneze ring, leading to the precursor molecules being connected together via single bonds. As the solvothermal treatment continues, reactions such as dehydrogenation and dealkylation would take place, resulting in the formation of large π‐conjugated structure which is N doped and highly surface passivated with —NR_2_ at edges.

**Figure 5:**
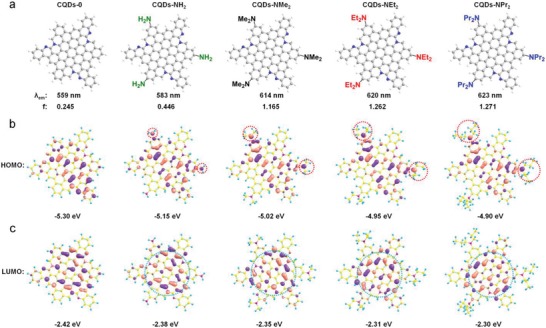
Time‐dependent density functional theory calculation results. a) The model CQDs structure, b) calculated HOMO, and c) LUMO of CQDs‐0 (without any EDG passivation), CQDs‐NH_2_, ‐NMe_2_,‐NEt_2_, and ‐NPr_2_, respectively (from left to right). Bottom rows of (a) are corresponding calculated λ_em_ and *f*.

Theoretical calculations were carried out to investigate the efficient red bandgap emissions of R‐EGP‐CQDs‐NMe_2_, ‐NEt_2_, and ‐NPr_2_. The optical properties of model CQDs consisting of the identical skeleton structure in Figure 1a without any EDGs (CQDs‐0) as well as with three ‐NH_2_, ‐NMe_2_, ‐NEt_2_, and ‐NPr_2_ (CQDs‐NH_2_, ‐NMe_2_, ‐NEt_2_, and ‐NPr_2_) at the edges were all calculated (**Figure** **5**a; Tables S5–S9, Supporting Information). It is found that all the five model CQDs exhibit rigid planar geometries as revealed by theoretical optimizations (Figure S9a–e, Supporting Information). The calculated absorption and emission spectral for CQDs‐0, ‐NH_2_, ‐NMe_2_, ‐NEt_2_, and ‐NPr_2_ are presented in Figure S10a–e of Supporting Information, and the calculated optical properties are listed in Table S10 of the Supporting Information. CQDs‐0 yields fluorescence emission (λ_em_) at 559 nm, but with a low calculated oscillator strength (*f*) of 0.245. However, a pronounced increase in *f* together with a red‐shift in λ_em_ is observed for CQDs‐NH_2_ (583 nm, 0.446), ‐NMe_2_ (614 nm, 1.165), ‐NEt_2_ (620 nm, 1.262), and ‐NPr_2_ (623 nm, 1.271), respectively. Moreover, the ‐NR_2_ passivation leads to much higher *f* and longer λ_em_ than —NH_2_, in good agreement of the experimental λ_em_ and QYs obtained from the reported —NH_2_ passivated CQDs (26%, 604 nm)^24^ and R‐EGP‐CQDs‐NMe_2_, ‐NEt_2_, and ‐NPr_2_ (77.9–86.0%, 637–645 nm). These results indicate that the red bandgap emission of R‐EGP‐CQDs‐NMe_2_, ‐NEt_2_, and ‐NPr_2_ originates from the rigid π‐conjugated skeleton structure, and the EDGs passivation does contribute to improve the QY.^39^ Then we analyzed the excited state characters of CQDs‐0, ‐NH_2_, ‐NMe_2_, ‐NEt_2_, and ‐NPr_2_ by examining their frontier orbitals (Figure 5b,c; Table S10, Supporting Information). It is reported that the presence of charge transfer (CT) excited state in a π‐conjugated system is beneficial to improve the QY and red‐shift the λ_em_.^40^, ^41^ The CT excited state can be qualitatively judged by the degree of spatial separation of the electron cloud density distributions in HOMO and LUMO. It is found that the HOMO and LUMO for CQDs‐0 are homogeneously distributed over the conjugated π‐surface. But from CQDs‐NH_2_ to ‐NPr_2_, the HOMOs are more distributed over the passivating EDGs owing to the increased electron‐donating ability from —NH_2_ to —NPr_2_ (red circles in Figure 5b), while the LUMOs are mainly distributed over the conjugated π‐surface (blue circles in Figure 5c), suggesting the presence of CT state. Hence it can be concluded that the CT excited state resulted from —NR_2_ passivation plays a key role in affording high QY of R‐EGP‐CQDs‐NMe_2_, ‐NEt_2_, and ‐NPr_2_ (Figures S11 and S12, Supporting Information).

The solubility of R‐EGP‐CQDs‐NMe_2_, ‐NEt_2_, and ‐NPr_2_ are evaluated in commonly used organic solvents at room temperature, and corresponding results are summarized in **Table** **1**. Due to the strong electronegativity of N and the presence of long pair electrons, ‐NR_2_ is classified as polar functional group and able to form hydrogen‐bond with protic solvents. Therefore, R‐EGP‐CQDs‐NMe_2_, ‐NEt_2_, and ‐NPr_2_ show high solubility in polar solvents, such as ethanol (14.0–14.6 mg mL^−1^), DMF (8.2–9.5 mg mL^−1^), and *o*‐dichlorobenzene (ODCB) (4.4–5.0 mg mL^−1^), with the help of —NR_2_ passivation.

**Table 1:** Solubility (mg mL^−1^) of R‐EGP‐CQDs‐NMe_2_, ‐NEt_2_, and ‐NPr_2_ in commonly used organic solvents at room temperature

Organic solvent	R‐EGP‐CQDs‐NMe_2_	R‐EGP‐CQDs‐NEt_2_	R‐EGP‐CQDs‐NPr_2_
EtOH	14.0	14.5	14.6
DMF	8.2	9.0	9.5
CH_3_CN	8.0	8.4	8.5
Chloroform	4.5	5.0	5.4
ODCB	4.4	4.8	5.0
Toluene	2.8	3.2	3.0
Cyclohexane	0.2	0.3	0.3

**Figure 6:**
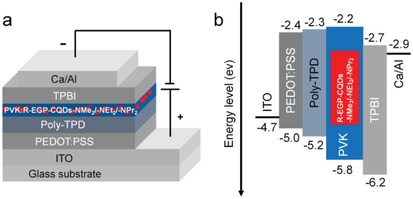
a) The device configuration and b) energy level diagram of WLEDs‐1, ‐2, and ‐3.

The merits of bright red bandgap emission and good organic solubility of R‐EGP‐CQDs‐NMe_2_, ‐NEt_2_, and ‐NPr_2_ have inspired us to explore their potentials in solution‐processed electroluminescent warm‐WLEDs. As shown in **Figure** **6**a, a full WLEDs configuration comprises, in the following order, a transparent indium‐tin‐oxide (ITO) anode, a poly(3,4 ethylenedioxythiophene): poly(styrenesulfonate) (PEDOT:PSS) hole injection layer (HIL), a poly(*N*,*N*9‐bis(4‐butylphenyl)‐*N*,*N*9‐bis(phenyl)‐benzidine) (poly‐TPD) hole transport layer (HTL), an active R‐EGP‐CQDs‐NMe_2_/‐NEt_2_/‐NPr_2_ blended poly(*N*‐vinyl carbazole) (PVK) EML, a 1,3,5‐tris(*N*‐phenylbenzimidazol‐2‐yl) benzene (TPBI) electron transport layer (ETL), and a Ca/Al double‐layered cathode. The thickness of PEDOT:PSS, poly‐TPD, PVK:R‐EGP‐CQDs (9 wt%), and TPBI layers in WLEDs devices are determined to be about 25, 22, 20, and 35 nm, respectively, as confirmed by the cross‐sectional TEM images and the EDX mapping images of WLEDs devices (Figures S13 and S14, Supporting Information). PVK is selected as host because it emits blue light, which is spectrally complementary to the red emission of R‐EGP‐CQDs‐NMe_2_, ‐NEt_2_, and ‐NPr_2_ for warm white light generation. Simultaneously, PVK is used to improve film forming ability and facilitate hole transport as well.^18^, ^42^ On the basis of the energy level diagram of WLEDs (Figure 6b), there exists small energy barriers for hole injection (0.6 eV) and electron injection (0.5 eV) from anode side and cathode side to the PVK host, respectively. Meanwhile, the HOMO and LUMO energy levels of R‐EGP‐CQDs‐NMe_2_, ‐NEt_2_, and ‐NPr_2_ are located within those of PVK host. Hence the electrons and holes can be smoothly injected to PVK host and effectively transferred to CQDs under proper bias voltage.^19^ Then the existed electrons and holes in the active EML would undergo radiative recombination to generate electroluminescence (EL).

**Figure 7:**
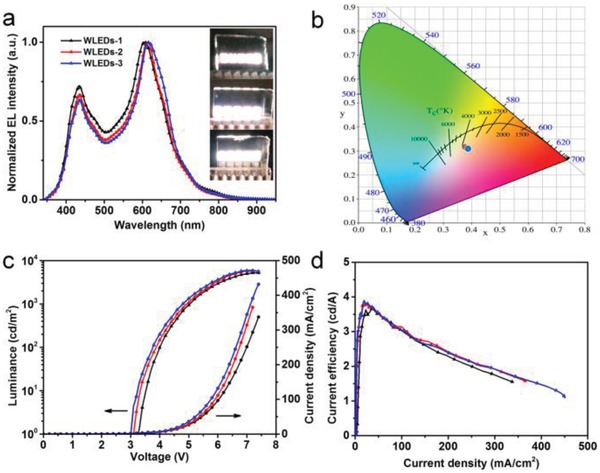
a) EL spectra, b) operation photographs (insets in (a), from top to bottom), and corresponding CIE coordinates of WLEDs‐1, ‐2, and ‐3 under 7.0 V, respectively. c) The *J*–*V*–*L* and d) current efficiency of WLEDs‐1 (black), ‐2 (red), and ‐3 (blue).

WLEDs‐1, ‐2, and ‐3 were fabricated with 9 wt% R‐EGP‐CQDs‐NMe_2_, ‐NEt_2_, and ‐NPr_2_ blended PVK as EML, respectively. The EL spectra under 7.0 V are shown in **Figure** **7**a. All three WLEDs generate warm white light with two independent peaks centered at around 434/605, 435/612, and 435/616 nm, respectively, which are attributed to the emission from PVK host and CQDs. Note that the EML is spin‐coated from ODCB solution, and the EL from CQDs is close to the PL in ODCB (591, 601, and 605 nm, Figure S15, Supporting Information). The photographs in the insets of Figure 7a display the close‐up view of the bright, uniform, and defect‐free surface warm white EL emission of WLEDs‐1, ‐2, and ‐3. The corresponding Commission Internationale de L'Eclairage (CIE) coordinate and CCT are (0.379, 0.314)/3365 K, (0.383, 0.311)/3168 K, and (0.388, 0.309)/2987 K (WLEDs‐3), respectively (Figure 7b). Meanwhile the shapes of EL spectra of WLEDs‐1, ‐2, and ‐3 almost do not change when varying the driving voltage from 4.0 to 7.0 V, which is likely due to the high CQDs concentration (9 wt%) in the EML. In this case, the carriers trapped by CQDs cannot be easily saturated with the voltage increasing, that is, no more excitons are formed on PVK to give off more blue emission relative to the red emission at high voltage.^43^

The performances of WLEDs‐1, ‐2, and ‐3 are summarized in **Table** **2** and Figure S11 (Supporting Information), and experimental current–voltage–luminance (*J*–*V*–*L*) and current efficiency (η_c_) and power efficiency (η_p_) curves are shown in Figure 7c,d and Figure S17 (Supporting Information). The turn‐on voltage (*V*_on_), defined as the bias voltage applied to LEDs producing a brightness of 1 cd m^−2^, is as low as 3.3–3.0 V. By taking advantage of the efficient red bandgap emission of R‐EGP‐CQDs‐NMe_2_, ‐NEt_2_, and ‐NPr_2_, the *L*_max_ and η_c,max_ reaches as high as about 5248–5909 cd m^−2^, 3.65–3.85 cd A^−1^, respectively. As far as we know, this is the first time to realize high performance electroluminescent warm‐WLEDs based on CQDs. The device performances are the best among all reported CQDs‐based WLEDs,^18^, ^31^, ^42^, ^44^, ^45^ and even comparable to the semiconductor Cd^2+^‐QDs‐based WLEDs (Table S12, Supporting Information).^14^, ^15^ The operation stability for WLEDs‐1, ‐2, and ‐3 are tested on 10 parallel devices (Figure S18, Supporting Information). After operation for 50 h, more than 80% of initial luminance (*L*_0_: 1000 cd m^−2^) are retained, demonstrating a long‐term device stability of WLEDs‐1, ‐2, and ‐3. Furthermore, the emission color could be tuned from warm white to red by increasing the CQDs concentration ratio from 9 to 30 wt% (Figure S19, Supporting Information). As shown in Figure S20a,b of the Supporting Information, we realized three monochrome red LED (RLEDs‐1, ‐2, and ‐3) with red EL spectra at 611, 621, and 626 nm, respectively, based on the same device configuration at the R‐EGP‐CQDs‐NMe_2_, ‐NEt_2_, and ‐NPr_2_ concentration ratio of 30 wt%. At this high CQDs concentration ratio, the PVK emission at 435 nm is quenched due to the complete energy transfer from PVK to CQDs.^19^ Bright red EL emission of RLEDs‐1, ‐2, and ‐3 are observed from the operation photographs shown in the insets of Figure S20a of the Supporting Information. They show stable EL spectra over bias voltage (Figure S21, Supporting Information), together with *L*_max_ and η_c,max_ as high as 2687–2960 cd m^−2^ and 2.11–2.19 cd A^−1^, respectively (Figure S20c,d and Table S13, Supporting Information).

**Table 2:** The performances of WLEDs‐1, ‐2, and ‐3

LEDs	EL peak [nm]	*V*_on_ ^a)^ [V]	*L*_max_ ^b)^ [cd m^−2^]	η_c,max_ ^c)^ [cd A^−1^]	CIE^d)^	CCT^e)^ [K]
WLEDs‐1	605/434	3.3	5248	3.65	(0.379, 0.314)	3365
WLEDs‐2	612/435	3.1	5802	3.81	(0.383, 0.311)	3168
WLEDs‐3	616/435	3	5909	3.85	(0.388, 0.309)	2987

^a)^ Turn‐on voltage corresponding to 1 cd m^−2^

^b)^ Maximum luminance

^c)^ Maximum current efficiency

^d)^ Obtained at maximum luminance

^e)^ Obtained at maximum luminance.

## 3 Conclusion

In summary, we successfully demonstrated the effectiveness of the electron‐donating group passivation strategy to impart R‐EGP‐CQDs‐NMe_2_, ‐NEt_2_, and ‐NPr_2_ with the ability of displaying red bandgap emissions at 637, 642, and 645 nm, respectively, with the highest QY up to 86.0% in ethanol. Theoretical investigations reveal that the red bandgap emission originates from the rigid π‐conjugated skeleton structure, and the ‐NR_2_ passivation plays a key role in inducing charge transfer excited state in the π‐conjugated structure to afford high QY. Moreover, the polar ‐NR_2_ groups help the R‐EGP‐CQDs‐NMe_2_, ‐NEt_2_, and ‐NPr_2_ with good solubility in common organic solvents. Solution‐processed electroluminescent warm‐WLEDs with the R‐EGP‐CQDs‐NMe_2_, ‐NEt_2_, and ‐NPr_2_ blended PVK as the active EML display voltage‐stable warm white spectrum, and high‐performance with maximum luminance of 5248–5909 cd m^−2^ and current efficiency of 3.65–3.85 cd A^−1^. The warm‐WLEDs also show good long‐term operation stability (*L*/*L*_0_ > 80% after 50 h operation, *L*_0_: 1000 cd m^−2^). Furthermore, monochrome red LEDs (RLEDs) are also realized by increasing the concentration ratio of CQDs in PVK, showing *L*_max_ and η_c,max_ up to 2687–2960 cd m^−2^ and 2.11–2.19 cd A^−1^, respectively. This work opens up a new avenue to realizing efficient red bandgap emission CQDs by passivating strong electron‐donating groups for the development of high‐performance electroluminescent warm‐WLEDs.

## 4 Experimental Section

*Material: N*,*N*‐dimethyl‐, *N*,*N*‐diethyl‐, and *N*,*N*‐dipropyl‐para‐phenylenediamine were purchased from Aladdin Industrial Corporation. Ethanol, chloroform, ODCB, etc. were purchased from Beijing Chemical Works. All chemicals were used without further purification unless otherwise specified.

*Synthesis of R‐EGP‐CQDs‐NMe_2_, ‐NEt_2_, and ‐NPr_2_*: R‐EGP‐CQDs were synthesized through a solvothermal method. In a typical preparation procedure for the synthesis of R‐EGP‐CQDs‐NMe_2_: a certain amount of starting material *N*,*N*‐dimethyl‐p‐PD (20 mg) was dissolved in DMF (10 mL) to form a 2 mg mL^−1^ clear solution after sonication for 2 min. Then the clear precursor solution was transferred to a 25 mL poly(tetrafluoroethylene) (Teflon)‐lined autoclave reactor and heated at 200 °C for 12 h in oven. After the reaction, the reactor was cooled to room temperature by water or naturally. A dark brown solution was obtained from the reactor, shining pink fluorescence (unpurified crude product containing blue or other colored fluorophors) under continuous 365 nm UV light, indicating the successful formation of R‐EGP‐CQDs‐NMe_2_. Purification process was carried out through silica column chromatography by using a mixture of methylene chloride and methanol (10:1 or less) as the eluent. After purification, a clear amaranth solution (dissolving in ethanol) was obtained, shining bright red fluorescence without any other color under continuous 365 nm UV light, indicating pure R‐EGP‐CQDs‐NMe_2_ was finally acquired. R‐EGP‐CQDs‐Et_2_, and ‐NPr_2_ were synthesized through the same procedure but the precursors were *N*,*N*‐diethyl‐ and *N*,*N*‐dipropyl‐p‐PD, respectively. The reaction conditions, such as precursor concentration, reaction temperature, reaction time, etc. were thoroughly optimized in this work.

*Measurement of HOMO and LUMO Energy Levels by Cyclic Voltammetry*: A conventional method of CV measurement was carried out to evaluate the HOMO and LUMO energy levels of R‐EGP‐CQDs‐NMe_2_, ‐NEt_2_, and ‐NPr_2_. In detail, a standard three electrode system was set up, composing of a glassy carbon electrode as the working electrode, a platinum wire as the counter electrode, and a Ag/Ag^+^ as the reference electrode. CV curves were recorded at a scan rate of 50 mV s^−1^ in acetonitrile containing 0.1 m (Bu)_4_NPF_6_ (supporting electrolyte) and R‐EGP‐CQDs‐NMe_2_, ‐NEt_2_, and ‐NPr_2_. During the whole CV measurement, high‐purity N_2_ was continuously pumped into the electrolyte to remove dissolved oxygen. The HOMO and LUMO levels were calculated from the onset potentials of first oxidation and first reduction and by assuming the energy level of ferrocene/ferrocenium (Fc/Fc^+^) to be −4.80 eV below the vacuum level. The formal potential of Fc/Fc^+^ was measured as 0.19 V against Ag/Ag^+^ using CVs measurement. The HOMO and LUMO energy levels extracted from CVs were calculated from the following equations

(1) $$E_{\mathrm{HOMO}}\;=\;-e(E_{\mathrm{ox}}\;+\;4.80)\;\mathrm{eV}$$

(2) $$E_{\mathrm{LUMO}}\;=\;-e(E_{\mathrm{red}}\;+\;4.80)\;\mathrm{eV}$$

*Theoretical Calculations*: The optical properties of five model CQDs were all calculated by using time‐dependent density functional theory method as carried out in the Gaussian09 software package. The 6–31G* basis set was selected to combine with the functional B3LYP throughout all calculations (B3LYP/6‐31G*). The first excited state was optimized in vacuum to calculate the emission energy (wavelength), which is the energy difference between the ground and the first excited state. The pink and violet colors in the HOMO and LUMO molecular orbitals represent the positive and negative phases of the molecular orbital wavefunctions.

*Device Fabrication and Characterization*: ITO‐coated glass substrates were cleaned ultrasonically in organic solvents (acetone and isopropyl alcohol), rinsed in deionized water, and then dried in an oven at 150 °C for 10 min. The substrates were cleaned with an UV‐ozone treatment to enrich the ITO surface with oxygen to increase the ITO work function. The PEDOT:PSS HIL (25 nm) was spin‐coated at 3000 rpm for 30 s on the ITO, followed by annealing in an oven at 150 °C for 15 min. A poly‐TPD HTL (20 nm) was then spin‐coated at 2000 rpm for 30 s over the surface of PEDOT:PSS film using 5 mg mL^−1^ poly‐TPD toluene solution, followed by annealing in an oven at 150 °C for 15 min. Afterward, the EML (20 nm) of 9 wt% R‐EGP‐CQDs‐NMe_2_, ‐NEt_2_, and ‐NPr_2_ blended PVK (5 mg mL^−1^) was spin‐coated at 2000 rpm for 30 s over the surface of poly‐TPD HTL using the mixed ODCB solution of R‐EGP‐CQDs (0.5 mg mL^−1^) and PVK (5 mg mL^−1^), followed by baking on a hot plate at 100 °C for 30 min to form the active region. Finally, the substrates were transferred to a vacuum chamber and a 40 nm thick TPBI ETL was thermally deposited with base pressure of 3 × 10^−4^ Pa. After that, a 20 nm Ca and 100 nm thick Al cathode was deposited using a shadow mask with 2 mm width. The active area of the devices was thus 4 mm^2^. The thermal deposition rates for TPBI and Ca/Al are 1, 1, and 3 Å s^−1^, respectively. PEDOT:PSS was used as a buffer layer on the anode mainly to increase the anode work function from 4.7 (ITO) to 5.0 eV and to reduce the surface roughness of the anode to obtain stable and pinhole free electrical conduction across the device. TPBI was chosen as the ETL because of its good electron transport capability and its interfacial phase compatibility with the EML. The thickness of films was measured using a Dektak XT (Bruker) surface profilometer and a spectroscopic ellipsometer (Suntech). The *J*–*V*–*L* characteristics were measured using a computer‐controlled Keithley 236 SMU and Keithley 200 multimeter coupled with a calibrated Si photodiode. EL spectra were measured by an Ocean Optics 2000 spectrometer, which couples a linear charge coupled device‐array detector ranging from 350 to 1100 nm.

*Characterization Method*: TEM and HRTEM images of R‐EGP‐CQDs were collected using JEOL JEM 2100. AFM measurements were performed with Veeco Dimension 3100 V. XRD patterns were carried out using Cu‐Kα radiation (XRD, PANalytical X'Pert Pro MPD). Raman spectrum was measured using Laser Confocal Micro‐Raman Spectroscopy (LabRAM Aramis) with 532 nm laser beam as the excitation source. UV–vis absorption spectra were recorded on UV‐2450 spectrophotometry. The PL emission and excitation spectra were measured on a PerkinElmer‐LS55 luminescence spectrometer. The photographs were taken with camera (Nikon, D7100) under UV light excited at 365 nm (UV light: SPECTROLINE, ENF‐280C/FBE, 8W). The absolute QY were determined by using Hamamatsu Photonics Quantaurus QY at room temperature. The FTIR spectra were measured using a Nicolet 380 spectrograph. XPS was performed with an ESCALab220i‐XL electron spectrometer from VG Scientific using 300 W Al Kα radiation.

## Conflict of Interest

The authors declare no conflict of interest.

## Supporting information

Supplementary
