# Supplementary material for: Electroluminescent Warm White Light‐Emitting Diodes Based on Passivation Enabled Bright Red Bandgap Emission Carbon Quantum Dots
Source: Adv Sci (Weinh). 2019 Apr 30;6(13):1900397. doi: 10.1002/advs.201900397 (PMC6662328; doi:10.1002/advs.201900397)
Supplement: Supplementary file 1 — Supplementary [file ADVS-6-1900397-s001.pdf]

Copyright WILEY-VCH Verlag GmbH & Co. KGaA, 69469 Weinheim, Germany, 2019.

ADVANCED SCIENCE
Open Access

Supporting Information

for *Adv. Sci.,* DOI: 10.1002/advs.201900397

Electroluminescent Warm White Light-Emitting Diodes Based on Passivation Enabled Bright Red Bandgap Emission Carbon Quantum Dots

Haoran Jia, Zhibin Wang, Ting Yuan, Fanglong Yuan, Xiaohong Li, Yunchao Li, Zhan'ao Tan, Louzhen Fan,* and Shihe Yang**

Copyright WILEY-VCH Verlag GmbH & Co. KGaA, 69469 Weinheim, Germany, 2019.

Supporting Information

Electroluminescent Warm White Light-Emitting Diodes Based on Passivation Enabled Bright Red Bandgap Emission Carbon Quantum Dots

Haoran Jia, Zhibin Wang, Ting Yuan, Fanglong Yuan, Xiaohong Li, Yunchao Li, Zhan'ao Tan, Louzhen Fan,* and Shihe Yang**

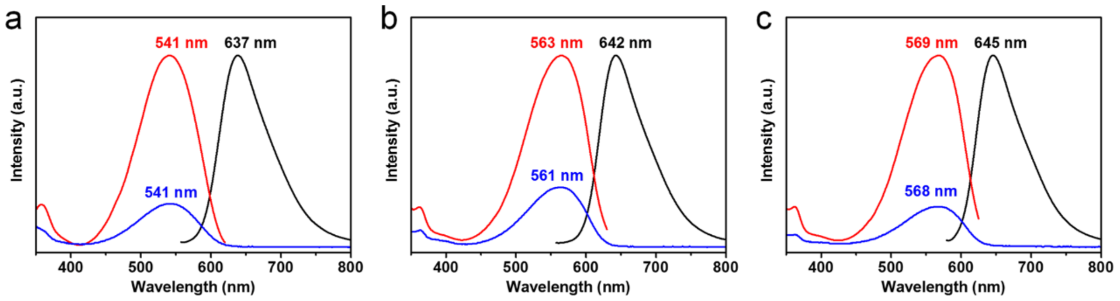

Figure S1. UV-vis absorption (blue line), normalized PL emission (black line, fixed excitation wavelength at 540 nm) and excitation (red line, fixed emission wavelength at 640 nm) spectra of R-EGP-CQDs-NMe_2 (a), -NEt_2 (b), and -NPr_2 (c) dilute ethanol solution.

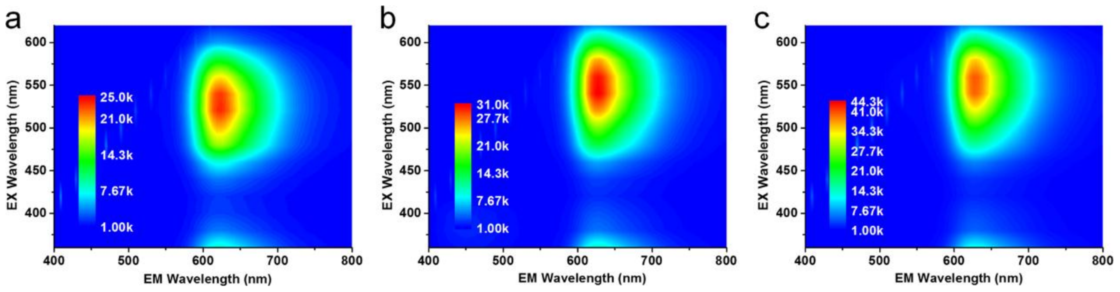

Figure S2. Excitation-Emission-Matrix Spectra of R-EGP-CQDs-NMe_2 (a), -NEt_2 (b), and -NPr_2 (c) dilute ethanol solution.

Table S1. Photoluminescence comparison of R-EGP-CQDs-NMe_2, -NEt_2, and -NPr_2 with other previously reported representative red-emissive CQDs.

Emission wavelength (nm)	Quantum yields (%)	Solubility	Reference	
637	77.9	Ethanol, DMF, chloroform, etc.	R-EGP-CQDs-NMe_2	This work
642	85.2		R-EGP-CQDs-NEt_2	
645	86.0		R-EGP-CQDs-NPr_2	
598	54	Ethanol	Nat. Commun. 2018, 9, 2249.5	
628	53	Ethanol	Adv. Mater. 2017, 29. 1702910.1	
640	48.7	Ethanol	Small 2018, 14, 1800612	
630	31.54	H_2O, Ethanol	Small 2018, 14, 1703919	
604	26.1	Ethanol	Angew. Chem., Int. Ed. 2015, 54, 5360-5363.3	
640	26	H_2O, DMSO, DMF, etc.	Adv. Mater. 2018, 30, 1705913	
640	22.9	Methanol	Chem. Mater. 2016, 28 (23), 8659-8668.4	
604	12	Ethanol	Adv. Mater. 2017, 29, 1604436	
610	5.0	H_2O	Chem. Commun. 2015, 51 (13), 2544-2546.2	
640	2.3	H_2O	Adv. Mater. 2015, 27, 4169-4177.7	

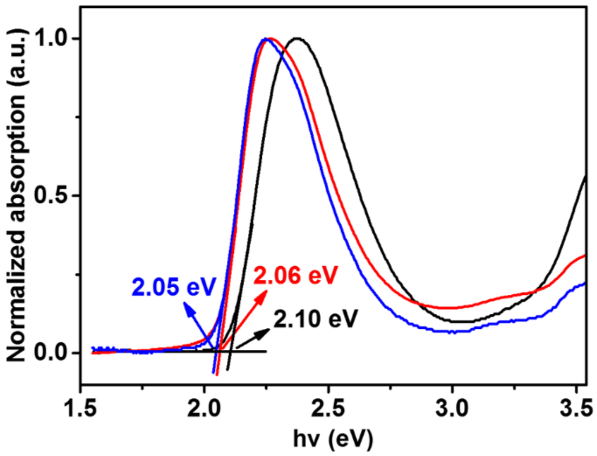

Figure S3. Absorption edges (Abs_{edge}) of R-EGP-CQDs-NMe_2 (black), -NEt_2 (red), and -NPr_2 (blue).

Table S2. Energy gaps of R-EGP-CQDs-NMe_2, -NEt_2, and -NPr_2 determined from CV curves and the absorption edges in dilute acetonitrile solution.

Sample	E_{Ox}^{onset} (V)	E_{Red}^{onset} (V)	HOMO (eV)	LUMO (eV)	$E_{g,CV}$ (eV)	Abs_{edge} (nm)	$E_{g,opt}$ (eV)
R-EGP-CQDs-NMe_2	0.12	-1.96	-4.92	-2.84	2.08	590	2.10
R-EGP-CQDs-NEt_2	0.02	-1.98	-4.82	-2.82	2.00	602	2.06
R-EGP-CQDs-NPr_2	-0.02	-1.99	-4.78	-2.81	1.97	605	2.05

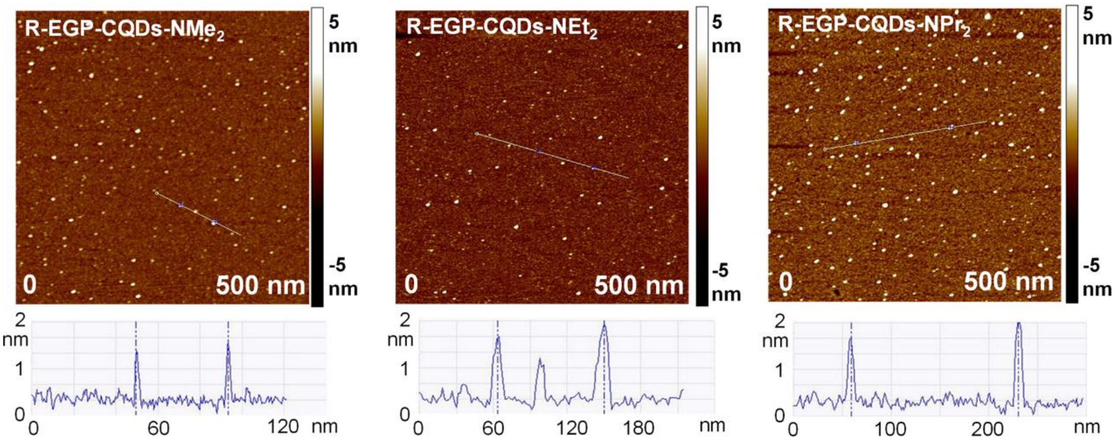

Figure S4. AFM images of R-EGP-CQDs-NMe_2, -NEt_2, and -NPr_2 (left to right).

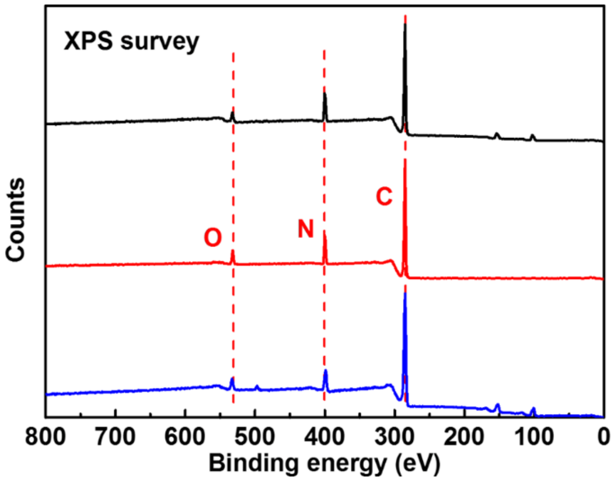

Figure S5. XPS survey spectra of R-EGP-CQDs-NMe_2 (black), -NEt_2 (red), and -NPr_2 (blue).

Table S3. Elemental analysis results of R-EGP-CQDs-NMe_2, -NEt_2, and -NPr_2 obtained from XPS survey spectra.

Sample	Peak Binding Energy (eV)			C : N : O
	C	N	O	
R-EGP-CQDs-NMe_2	284.8	399.8	531.2	7.5 : 1 : 0.31
R-EGP-CQDs-NEt_2	284.9	400.0	531.1	9.3 : 1 : 0.42
R-EGP-CQDs-NPr_2	284.8	399.9	531.4	11.2 : 1 : 0.38

Table S4. Detailed elemental analysis results of R-EGP-CQDs-NMe_2, -NEt_2, and -NPr_2 obtained from high-resolution C1s and N1s spectra.

Sample	C			N		
	C=C/C-C	C-N	C-O /C=O	pyridine N	tertiary amine	graphitic N
R-EGP-CQDs-NMe_2	284.5 (81.6%)	285.4 (16.1%)	288.0 (2.30%)	398.4 (8.10%)	399.9 (78.0%)	401.1 (13.9%)
R-EGP-CQDs-NEt_2	284.4 (81.5%)	285.4 (15.8%)	288.0 (2.70%)	398.4 (6.30%)	399.9 (78.1%)	401.0 (15.6%)
R-EGP-CQDs-NPr_2	284.2 (82.8%)	285.5 (15.3%)	287.9 (1.90%)	398.5 (9.20%)	399.9 (77.5%)	400.9 (13.3%)

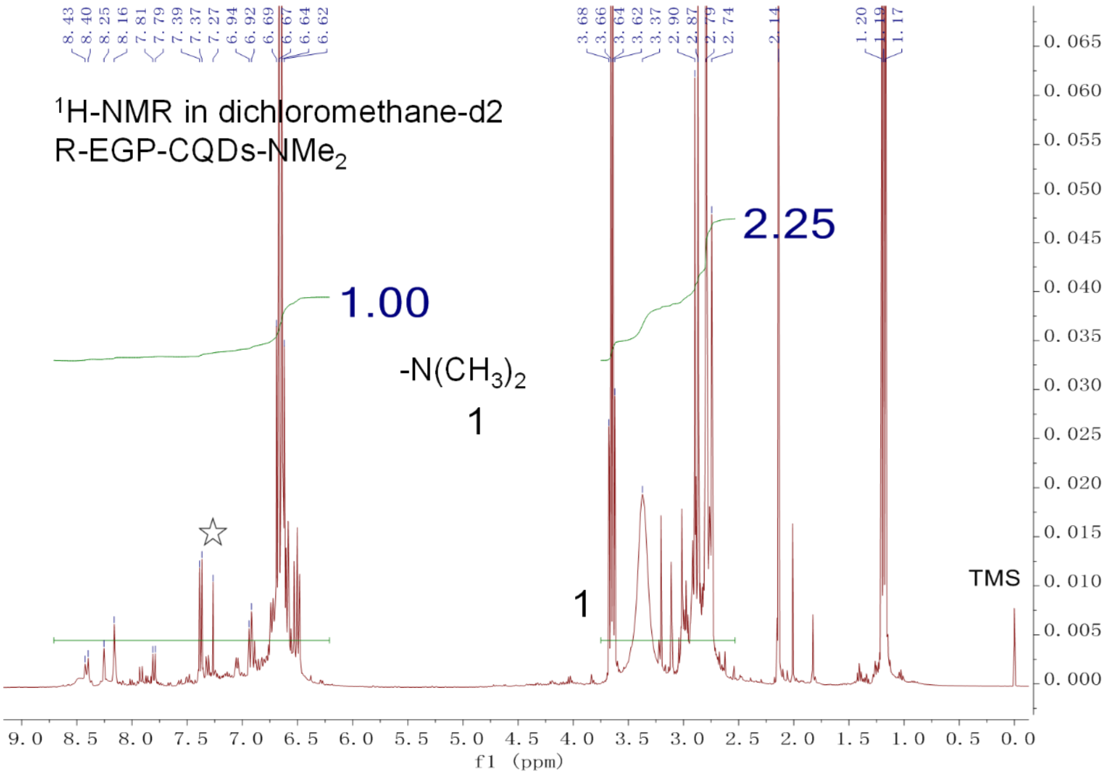

Figure S6. The integral processing of ^{1}H-NMR spectra of R-EGP-CQDs-NMe_2 in dichloromethane-d2.

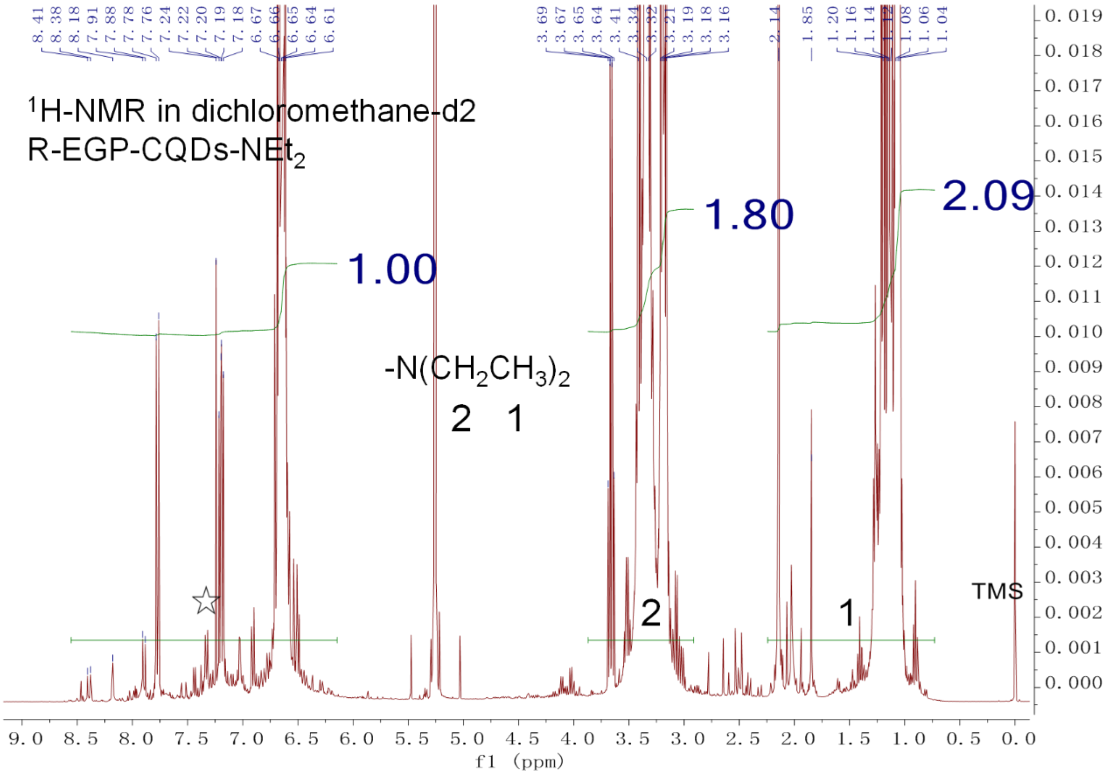

Figure S7. The integral processing of ^{1}H-NMR spectra of R-EGP-CQDs-NEt$_2$ in dichloromethane-d2.

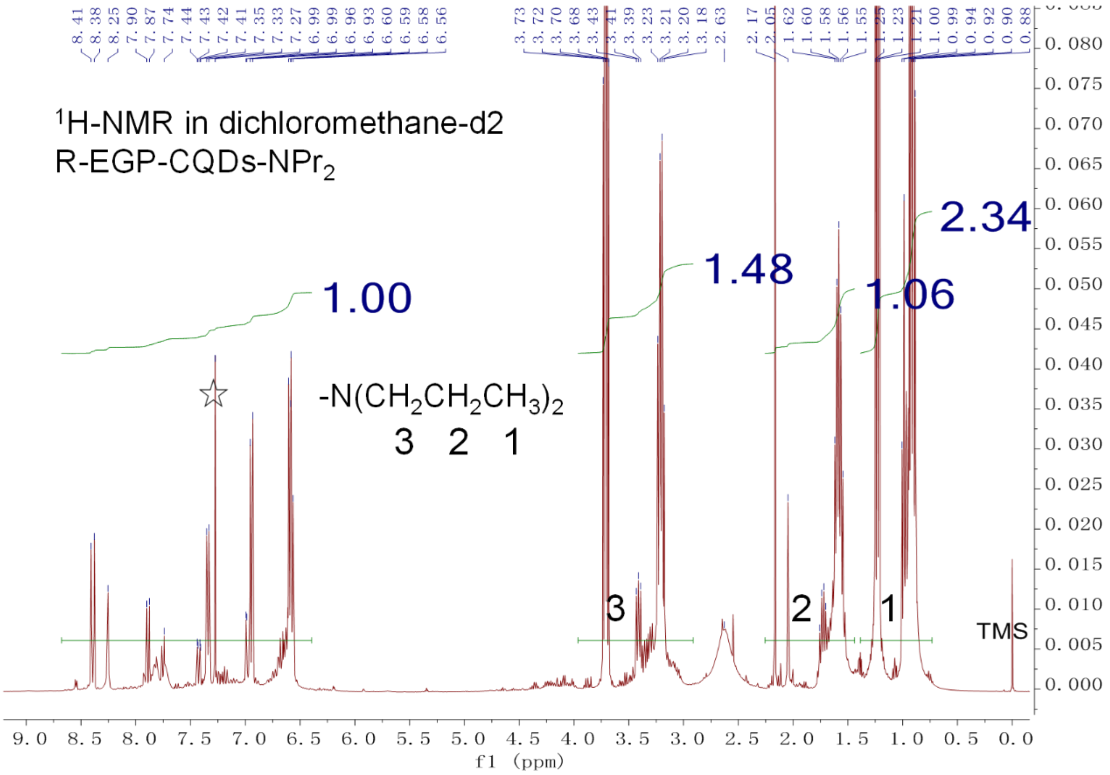

Figure S8. The integral processing of ^{1}H-NMR spectra of R-EGP-CQDs-NPr$_2$ in dichloromethane-d2.

Table S5. Optimized Cartesian coordinates (Å) of CQDs-0 for the first excited state.

C	-0.13510500	-3.75932200	0.00011100
C	-1.01786600	-2.65302300	0.00007800
C	-0.50547100	-1.32970100	0.00007300
C	0.89376200	-1.11025800	0.00008800
C	1.77406800	-2.22873900	0.00008200
C	1.26656500	-3.54846800	0.00011100
C	-1.40836800	-0.21889000	0.00005200
C	1.40444800	0.22698500	0.00006700
C	-2.81710500	-0.42191300	0.00003600
C	-3.70643700	0.67735500	0.00000800
C	-3.18807400	1.99662600	-0.00001400
C	-1.78867800	2.20794500	0.00000700
C	-0.89869900	1.10261500	0.00003800
C	0.51467100	1.32906600	0.00005800
C	1.04320600	2.65062200	0.00003300
C	2.43978700	2.87108800	0.00006600
C	3.32338300	1.76257700	0.00008500
C	2.80662400	0.44495500	0.00006900
N	-4.04017700	3.09162400	-0.00002300
C	2.18430400	-4.67113100	0.00001700
C	3.19532900	-2.00749700	0.00002200
C	3.69442800	-0.68126200	0.00003000
C	5.08633800	-0.44980300	0.00000600
C	6.00417100	-1.52944200	-0.00003400
C	5.48809000	-2.83350200	-0.00009300
C	4.11642600	-3.09878600	-0.00007100
C	3.59076400	-4.46715700	-0.00013300
C	4.43417200	-5.59616600	-0.00040700
C	3.91334400	-6.88057100	-0.00048100
C	2.52803800	-7.07327700	-0.00028100
C	1.66947500	-5.98124200	-0.00001200
C	-3.33619300	-1.76336600	0.00003200
C	-5.13745200	0.44403800	0.00003600
C	0.14081200	3.77086000	-0.00002200
C	2.95314000	4.22716100	-0.00004000
C	-1.25720000	3.53982700	-0.00001900
C	-2.15375700	4.62953900	-0.00005600
C	-1.67763600	5.96405400	-0.00008400
C	-0.29032400	6.16937500	-0.00011500
C	0.62533500	5.11413400	-0.00010500
C	2.07311100	5.34315200	-0.00017500
C	2.62916000	6.63803300	-0.00039600
C	4.00186900	6.82927900	-0.00042000
C	4.86151700	5.72598700	-0.00029000
C	4.34500300	4.43648600	-0.00007200

C	-5.66400700	-0.87602500	0.00008300
C	-7.06339500	-1.04185800	0.00013500
C	-7.91547000	0.05127700	0.00014300
C	-7.38980300	1.34739900	0.00012100
C	-6.01479800	1.54484100	0.00005000
C	-2.43703300	-2.85865100	0.00002800
C	-2.93261700	-4.17984300	-0.00000600
C	-4.32637700	-4.43482500	-0.00002700
C	-5.19786800	-3.33592200	-0.00001900
C	-4.74170200	-2.01539500	0.00002600
C	-5.50607900	2.95989000	-0.00006400
C	-3.57189800	4.40372200	-0.00006500
N	-4.47096900	5.36138100	-0.00006300
C	-4.03569200	6.66835900	-0.00003300
C	-5.02148100	7.68289200	0.00000600
C	-4.66569600	9.01943600	-0.00000100
C	-3.30667000	9.38668100	0.00002000
C	-2.32517100	8.40910700	-0.00005600
C	-2.65972100	7.03725900	-0.00006300
N	4.69777600	1.95296200	0.00009100
N	-0.65745000	-5.04482600	0.00007800
C	0.18970200	-6.24831600	0.00035000
C	-2.02791900	-5.29519600	0.00002000
C	5.59997400	0.89127400	0.00006900
C	5.31625500	3.28856400	0.00030100
N	6.87887700	1.19098000	0.00017200
C	-3.75747200	-6.82916100	0.00003400
C	-4.14344600	-8.19013600	0.00008100
C	-5.47884400	-8.55014800	0.00004500
C	-6.47626700	-7.55672100	-0.00003900
C	-6.12022400	-6.21799900	-0.00007000
C	-4.76485900	-5.82186700	-0.00003200
N	-2.40787900	-6.55262900	0.00008000
C	7.79310100	0.16055600	0.00013500
C	9.16451400	0.50734800	0.00025700
C	10.14436400	-0.46874800	0.00023000
C	9.78311200	-1.82935800	0.00008900
C	8.44585000	-2.19090400	-0.00001800
C	7.42468200	-1.21552700	0.00002000
H	6.18463000	-3.65983700	-0.00010900
H	5.51076300	-5.47626500	-0.00059700
H	4.58147100	-7.73654700	-0.00071500
H	2.11852500	-8.08039900	-0.00033700
H	0.07665400	7.18597800	-0.00015200
H	1.98696500	7.51045000	-0.00058300
H	4.40895200	7.83595700	-0.00056400
H	5.93850600	5.87462200	-0.00029900

H	-7.49762600	-2.03428000	0.00017100
H	-8.99079700	-0.09956100	0.00020500
H	-8.05702100	2.20581500	0.00012500
H	-6.26182100	-3.52608100	-0.00005700
H	-5.88890800	3.50393500	0.87113600
H	-5.88885400	3.50378200	-0.87139400
H	-6.06314900	7.37574900	0.00001700
H	-5.43532000	9.78646700	0.00000000
H	-3.02530500	10.43571600	0.00006000
H	-1.28327700	8.71289200	-0.00004800
H	-0.08986400	-6.85163100	0.87175000
H	-0.09016500	-6.85221800	-0.87052300
H	5.97893900	3.34844900	-0.87075900
H	5.97831600	3.34846800	0.87185000
H	-3.35694700	-8.93903100	0.00013400
H	-5.75848000	-9.60013200	0.00007200
H	-7.52546100	-7.83748500	-0.00006800
H	-6.90437700	-5.46772400	-0.00010900
H	9.41906600	1.56312500	0.00037000
H	11.19338700	-0.18554400	0.00032100
H	10.55111100	-2.59735400	0.00006100
H	8.18872000	-3.24524500	-0.00012300

Table S6. Optimized Cartesian coordinates (Å) of CQDs-NH_2 for the first excited state.

C	-0.84202500	3.66405900	0.00165900
C	0.29656500	2.82571600	0.00293400
C	0.14430300	1.41438700	0.00368600
C	-1.14975100	0.84110300	0.00315900
C	-2.28915700	1.69286000	0.00159100
C	-2.14160700	3.09849700	0.00177100
C	1.30329000	0.57516900	0.00501200
C	-1.29720000	-0.58217100	0.00305600
C	2.61052600	1.13598800	0.00613500
C	3.75428000	0.30554900	0.00749200
C	3.59412600	-1.10276300	0.00538300
C	2.29895200	-1.66965200	0.00382700
C	1.15270800	-0.83227400	0.00415200
C	-0.15351900	-1.41624500	0.00347300
C	-0.32155100	-2.82888300	0.00186900
C	-1.61262400	-3.40394500	0.00187900
C	-2.75224100	-2.56112400	0.00363800
C	-2.59549000	-1.15598700	0.00312600
N	4.70416200	-1.93938800	0.00403500
C	-3.31622600	3.94196600	-0.00037800
C	-3.60599700	1.11331700	0.00021900

C	-3.74496800	-0.29754500	0.00177200
C	-5.02886700	-0.88287800	0.00256600
C	-6.19504800	-0.07736600	0.00161900
C	-6.03413500	1.31590500	-0.00089200
C	-4.77822500	1.92931800	-0.00239200
C	-4.62552900	3.38952100	-0.00445600
C	-5.73153600	4.25453800	-0.01383300
C	-5.57615400	5.64368000	-0.01402300
C	-4.27045400	6.17554000	-0.00842600
C	-3.16564100	5.34144900	-0.00256500
C	2.76717900	2.56627300	0.00544400
C	5.07198100	0.90088300	0.01045100
C	0.83897800	-3.67956100	-0.00033400
C	-1.75617500	-4.84280300	-0.00265100
C	2.13024100	-3.09439700	0.00080400
C	3.27943300	-3.91358300	-0.00176200
C	3.16469300	-5.32622500	-0.00604700
C	1.87750900	-5.88368600	-0.00788000
C	0.71811900	-5.10274100	-0.00463800
C	-0.62292200	-5.70035900	-0.00721500
C	-0.81939100	-7.09061400	-0.00936200
C	-2.10010000	-7.65073500	-0.01224200
C	-3.21364500	-6.78588900	-0.00715500
C	-3.04348400	-5.41203700	-0.00132500
C	5.24843800	2.31099600	0.01043600
C	6.55072000	2.83616900	0.01031400
C	7.67607700	2.00700500	0.01362700
C	7.48357900	0.61026100	0.01356800
C	6.20875500	0.07073200	0.01156000
C	1.61461800	3.39194100	0.00316000
C	1.74952700	4.79663800	0.00111100
C	3.03013700	5.40352300	0.00148600
C	4.15635500	4.56774000	0.00458600
C	4.05998400	3.17324400	0.00624200
C	6.08495400	-1.42911600	0.00767400
C	4.59153300	-3.32713400	-0.00006200
N	5.70817300	-4.02035700	-0.00224800
C	5.62580100	-5.39554500	-0.00660100
C	6.84069900	-6.12058900	-0.00909900
C	6.84339800	-7.50384100	-0.01356300
C	5.62606200	-8.21054000	-0.01563100
C	4.42485500	-7.51993400	-0.01314200
C	4.39225900	-6.10850900	-0.00867500
N	-4.03172400	-3.10387700	0.00484600
N	-0.67253100	5.04365700	-0.00047100
C	-1.80490600	5.98444200	0.00098100
C	0.58555200	5.63974000	-0.00110600

C	-5.17697800	-2.31242200	0.00626100
C	-4.28092700	-4.55471300	0.01360900
N	-6.33546700	-2.93285600	0.01015800
C	1.86018000	7.56938400	-0.00375600
C	1.88059400	8.98400700	-0.00655900
C	3.07706900	9.67820200	-0.00689500
C	4.29758300	8.97715800	-0.00442100
C	4.30032700	7.59152400	-0.00173200
C	3.09427600	6.85754600	-0.00128200
N	0.62767200	6.95335200	-0.00326400
C	-7.48522200	-2.17393700	0.00880700
C	-8.72038700	-2.86379200	0.01298500
C	-9.91981800	-2.17474600	0.01219700
C	-9.92328300	-0.76722800	0.00713400
C	-8.72478600	-0.07184900	0.00327800
C	-7.48600300	-0.74917000	0.00429800
N	-6.67868700	6.48910400	0.04372700
N	8.95891900	2.53881000	0.08044200
N	-2.28112500	-9.02766300	-0.08208100
H	-6.92139000	1.93324400	-0.00012000
H	-6.74098200	3.85897300	-0.02019200
H	-4.13364300	7.25489100	-0.00855900
H	1.78699500	-6.96099700	-0.01252600
H	0.02789600	-7.76685700	-0.00957100
H	-4.21666400	-7.20714800	-0.00775700
H	6.71327600	3.90803300	0.00869000
H	8.34987400	-0.04778000	0.01590100
H	5.13422800	5.02821000	0.00601700
H	6.59510900	-1.85528200	0.87911500
H	6.59881600	-1.85153800	-0.86347600
H	7.76783500	-5.55476000	-0.00739400
H	7.78560300	-8.04521700	-0.01528600
H	5.62587700	-9.29671600	-0.01934600
H	3.49690700	-8.08293800	-0.01495700
H	-1.69495000	6.63764600	0.87429400
H	-1.69202200	6.64329900	-0.86750900
H	-4.91720300	-4.78801300	-0.84763800
H	-4.89223600	-4.78221300	0.89475200
H	0.92647100	9.50333700	-0.00835300
H	3.07480900	10.76486300	-0.00905300
H	5.23843200	9.51987300	-0.00465300
H	5.25250400	7.07009500	0.00000200
H	-8.69332600	-3.94965200	0.01675400
H	-10.85973300	-2.72003700	0.01538100
H	-10.86395800	-0.22416800	0.00628200
H	-8.74905900	1.01342900	-0.00078000
H	-6.52847600	7.42193200	-0.32228200

H	-7.54616200	6.09292000	-0.29877500
H	9.69444400	1.94292600	-0.28106500
H	9.05169100	3.48902000	-0.25908000
H	-3.16394800	-9.36729200	0.28098400
H	-1.50420500	-9.58420600	0.25459100

Table S7. Optimized Cartesian coordinates (Å) of CQDs-NMe_2 for the first excited state.

C	-3.71279700	0.58508900	-0.01191800
C	-2.79773800	-0.49235600	-0.01036800
C	-1.40046400	-0.24222400	-0.00578100
C	-0.91854100	1.08855300	-0.00312000
C	-1.84716700	2.16580300	-0.00469400
C	-3.23898000	1.92077200	-0.01043300
C	-0.48266900	-1.33976600	-0.00507600
C	0.49073800	1.33434700	0.00156700
C	-0.95137700	-2.68239100	-0.00811200
C	-0.04328600	-3.76535800	-0.00892800
C	1.35039400	-3.50803500	-0.00368600
C	1.82623900	-2.17703200	0.00011900
C	0.91082000	-1.09190700	-0.00066600
C	1.40246200	0.25150700	0.00264100
C	2.79960200	0.51686100	0.00720800
C	3.28324800	1.84474800	0.01098300
C	2.36308500	2.92306300	0.00822600
C	0.97258300	2.66956400	0.00398400
N	2.26122900	-4.55857100	-0.00225500
C	-4.16128300	3.03255900	-0.01178300
C	-1.36180600	3.52040500	-0.00171000
C	0.03622000	3.75674300	0.00209900
C	0.53187500	5.07794900	0.00374300
C	-0.35338200	6.18481400	0.00081700
C	-1.73235300	5.92763900	-0.00334800
C	-2.25861400	4.63259500	-0.00300800
C	-3.70627200	4.37777300	-0.00605600
C	-4.64374300	5.42160200	-0.00079000
C	-6.03123900	5.18252700	-0.01017600
C	-6.45859200	3.82983200	-0.00877400
C	-5.54633400	2.78955900	-0.01288900
C	-2.36725100	-2.93904100	-0.01175400
C	-0.54534000	-5.11963100	-0.01272600
C	3.73015300	-0.58102100	0.00806300
C	4.70721700	2.08709400	0.01531800
C	3.23576700	-1.90996800	0.00485100
C	4.13202800	-3.00003100	0.00591700

C	5.53313900	-2.78725200	0.01091100
C	6.00007700	-1.46450600	0.01480900
C	5.14179900	-0.36097000	0.01207700
C	5.64480800	1.02028600	0.01452000
C	7.01756100	1.31038900	0.01253300
C	7.50402200	2.63150100	0.02029200
C	6.54613300	3.67808600	0.01524400
C	5.18926600	3.40808800	0.01517700
C	-1.93787000	-5.39757900	-0.01350300
C	-2.37392500	-6.73093600	-0.01060100
C	-1.47365900	-7.81334400	-0.01762100
C	-0.08825200	-7.50801700	-0.01002600
C	0.35731000	-6.19799800	-0.01007600
C	-3.27111500	-1.84658700	-0.01290500
C	-4.66321500	-2.07780700	-0.01764600
C	-5.17910400	-3.39771200	-0.02243900
C	-4.26676900	-4.46310200	-0.02203700
C	-2.88223400	-4.27142600	-0.01506200
C	1.84612000	-5.97160900	-0.00425500
C	3.63760600	-4.34983300	0.00278700
N	4.40724000	-5.41562600	0.00343600
C	5.77345600	-5.23745000	0.00749000
C	6.58139300	-6.39872000	0.00732600
C	7.96158900	-6.30549700	0.01092800
C	8.58182900	-5.04198000	0.01473400
C	7.80922500	-3.89176500	0.01494100
C	6.39893800	-3.95730100	0.01145900
N	2.81707100	4.23723100	0.00947100
N	-5.07789600	0.32126100	-0.01534800
C	-6.09413800	1.38666600	-0.01753900
C	-5.58541800	-0.97492200	-0.01901800
C	1.94813500	5.32477900	0.00688900
C	4.24826500	4.58407000	0.01184400
N	2.48607900	6.52439200	0.00687400
C	-7.42139700	-2.38121500	-0.02849300
C	-8.83098300	-2.50082000	-0.03400000
C	-9.44033600	-3.74271200	-0.03875400
C	-8.65630400	-4.91148900	-0.03803800
C	-7.27385500	-4.81711900	-0.03274200
C	-6.62555400	-3.56283700	-0.02802200
N	-6.89331200	-1.10865300	-0.02417200
C	1.64848600	7.61799000	0.00482100
C	2.25054500	8.89793700	0.00577700
C	1.48029800	10.04702000	0.00463100
C	0.07594000	9.95292100	0.00246000
C	-0.53414100	8.70869700	0.00109500
C	0.22715100	7.51983200	0.00205100

N	-6.94373400	6.21620600	-0.02727900
N	-1.91491500	-9.11981600	-0.03796600
N	8.85583700	2.90435000	0.03924300
C	-0.96234800	-10.21324200	0.09738300
C	-3.33730900	-9.40433700	0.08422500
C	9.32439300	4.27640500	-0.10013400
C	9.81527600	1.81633600	-0.08232900
C	-8.36693100	5.93425600	0.10207600
C	-6.48345400	7.59185800	0.09549200
H	-2.40850000	6.77106800	-0.00913400
H	-4.29543400	6.44355200	0.01307000
H	-7.51612700	3.59092200	-0.00371500
H	7.06866900	-1.30076200	0.02148700
H	7.72847600	0.49792400	0.00213900
H	6.86815200	4.71328400	0.01018300
H	-3.43330300	-6.93981400	-0.00115600
H	0.64676700	-8.30493600	-0.00259400
H	-4.65898800	-5.47038200	-0.03005300
H	2.30855500	-6.45332800	-0.87380500
H	2.30168900	-6.45384100	0.86858100
H	6.08058300	-7.36258100	0.00422000
H	8.56710300	-7.20780700	0.01069100
H	9.66533200	-4.96615100	0.01730700
H	8.30608400	-2.92697300	0.01736500
H	-6.73659700	1.23039200	-0.89192000
H	-6.74445900	1.22709500	0.85036700
H	4.43424300	5.22334700	0.88258500
H	4.43798600	5.22158900	-0.85945700
H	-9.41586700	-1.58565000	-0.03436500
H	-10.52452000	-3.81564800	-0.04293400
H	-9.13204800	-5.88789900	-0.04162100
H	-6.68703000	-5.73013500	-0.03193900
H	3.33564300	8.94589100	0.00775600
H	1.95964100	11.02221600	0.00558300
H	-0.53138500	10.85342400	0.00194200
H	-1.61817100	8.65709000	-0.00010100
H	-0.22455300	-10.20191100	-0.71397700
H	-0.41953600	-10.18078600	1.05359300
H	-1.49960000	-11.16052900	0.04307300
H	-3.90555400	-8.94579200	-0.73458200
H	-3.49142100	-10.48269500	0.03348700
H	-3.75570700	-9.04103200	1.03464200
H	8.94691200	4.91109400	0.71068800
H	10.41346900	4.28582800	-0.04857200
H	9.02185500	4.72776300	-1.05654800
H	9.70602800	1.09664000	0.73847800
H	9.70964300	1.26984400	-1.03129100

H	10.82534500	2.22471700	-0.03447500
H	-8.61310700	5.44643400	1.05679400
H	-8.92118400	6.87170500	0.04669500
H	-8.72160600	5.28950100	-0.71124300
H	-5.80538600	7.85913900	-0.72454300
H	-7.34308000	8.26104500	0.04770000
H	-5.95811400	7.77371200	1.04474800

Table S8. Optimized Cartesian coordinates (Å) of CQDs-NEt_2 for the first excited state.

C	1.03635507	3.60911461	-0.09892357
C	1.62640363	2.32465127	-0.08706734
C	0.80903754	1.16446340	-0.05628513
C	-0.60056606	1.28972437	-0.03719046
C	-1.18481822	2.58616417	-0.05138656
C	-0.37428712	3.74314897	-0.08453755
C	1.41599034	-0.13090081	-0.04585770
C	-1.41867352	0.11682977	-0.00417293
C	2.83056904	-0.27357384	-0.06550870
C	3.42711303	-1.55430120	-0.05887273
C	2.60589530	-2.70846334	-0.02610885
C	1.19883480	-2.57722399	-0.00357191
C	0.60270467	-1.28897509	-0.01418521
C	-0.82220765	-1.16683717	0.00789235
C	-1.65280625	-2.32052096	0.04139620
C	-3.05982744	-2.19645289	0.06508405
C	-3.64920179	-0.90790763	0.04875789
C	-2.83208073	0.24491588	0.01499260
N	3.17398830	-3.97725175	-0.01376683
C	-0.99313828	5.04755752	-0.10135991
C	-2.61724630	2.71947844	-0.03463276
C	-3.42206429	1.55255865	-0.00229849
C	-4.82887595	1.66105090	0.01208064
C	-5.45804205	2.93090995	-0.00910979
C	-4.64261969	4.07186639	-0.04415934
C	-3.24627630	4.00246520	-0.05344698
C	-2.40211873	5.20526425	-0.08623682
C	-2.95154310	6.49830868	-0.09370475
C	-2.15587749	7.65879704	-0.14190932
C	-0.74885927	7.46590838	-0.14071385
C	-0.18963134	6.20154698	-0.12458867
C	3.66235596	0.90065996	-0.09301640
C	4.86610637	-1.67204550	-0.07785410
C	-1.05163105	-3.62832296	0.05208228
C	-3.88096662	-3.38386116	0.10072305

C	0.36142506	-3.74167755	0.02994434
C	0.97144339	-5.01433300	0.04067181
C	0.18674799	-6.19383920	0.07380440
C	-1.20950793	-6.05806248	0.09529791
C	-1.84770737	-4.81448675	0.08330630
C	-3.31257436	-4.68514154	0.10565091
C	-4.15659681	-5.80448148	0.13030762
C	-5.56107968	-5.69614990	0.16930114
C	-6.09739670	-4.38225321	0.14586620
C	-5.27944164	-3.26489563	0.11965927
C	5.70844655	-0.52927079	-0.09518034
C	7.09998697	-0.70000353	-0.09727307
C	7.70971405	-1.97086810	-0.10197686
C	6.83995843	-3.09238748	-0.06879937
C	5.46313855	-2.94276795	-0.06473420
C	3.05376599	2.18128118	-0.10393620
C	3.85093201	3.34544576	-0.13181989
C	5.26496303	3.25530970	-0.14936359
C	5.84568205	1.97819001	-0.13808924
C	5.08777516	0.80411120	-0.10812142
C	4.63002675	-4.19759432	-0.04019996
C	2.40380153	-5.13636395	0.01826882
N	3.04483597	-6.28403947	0.02572733
C	2.30616492	-7.44654928	0.05827325
C	3.01769356	-8.66924025	0.06631055
C	2.35081785	-9.88075780	0.09845906
C	0.94386277	-9.90932004	0.12376628
C	0.22726121	-8.72339561	0.11624507
C	0.88176632	-7.47276954	0.08374554
N	-5.03188414	-0.76492699	0.06458521
N	1.85130689	4.73507360	-0.12627674
C	1.31410785	6.10660369	-0.12978405
C	3.24045929	4.64716338	-0.14233136
C	-5.65088170	0.48188958	0.04522427
C	-5.95000771	-1.91601545	0.10077205
N	-6.96527781	0.50110665	0.05847376
C	5.29049103	5.71678845	-0.18370424
C	5.99427672	6.94375157	-0.20974254
C	7.37717970	6.97098986	-0.22961547
C	8.10529362	5.76650338	-0.22370333
C	7.43597357	4.55351977	-0.19798363
C	6.02538991	4.49612617	-0.17753379
N	3.91406605	5.77576684	-0.16553302
C	-7.60259233	1.72246641	0.03955843
C	-9.01719292	1.71822343	0.05488837
C	-9.73302479	2.90180464	0.03871150
C	-9.05429470	4.13439581	0.00708652

C	-7.66900592	4.16142151	-0.00866410
C	-6.91314840	2.96894419	0.00630903
N	-2.71216994	8.93236575	-0.21505746
N	9.09014492	-2.12589196	-0.15780216
N	-6.38460543	-6.81496623	0.24974781
C	9.64062743	-3.48724344	-0.04504133
C	9.95764138	-0.99068783	0.17299550
C	-7.84155390	-6.60737936	0.19930016
C	-5.85936166	-8.12619416	-0.14640732
C	-1.91041978	10.09453763	0.18613147
C	-4.17773979	9.04657988	-0.15242476
C	11.13655325	-3.60997967	-0.33189115
C	10.03492004	-0.66509293	1.67057653
C	-5.70009164	-8.31378491	-1.66079780
C	-8.68333049	-7.83595679	0.54318033
C	-4.73573970	10.43009030	-0.48389186
C	-1.70963042	10.23374315	1.70083069
H	-5.12219288	5.04018643	-0.07044262
H	-4.02362505	6.60275372	-0.04466755
H	-0.07825929	8.31664146	-0.15819317
H	-1.80834242	-6.95746186	0.12493875
H	-3.71126182	-6.78712601	0.12429675
H	-7.16850505	-4.22429622	0.13386039
H	7.72697925	0.17785170	-0.09604698
H	7.23911333	-4.09830818	-0.03475140
H	6.92425896	1.90904252	-0.15664732
H	4.88912187	-4.81196882	0.82983335
H	4.85566260	-4.82273446	-0.91225986
H	4.10259577	-8.62248882	0.04657820
H	2.91320760	-10.81053630	0.10426418
H	0.41783036	-10.85927490	0.14911659
H	-0.85704504	-8.76703564	0.13557793
H	1.73334084	6.63006450	0.73759883
H	1.72678123	6.62177648	-1.00461033
H	-6.61990261	-1.83476371	-0.76320354
H	-6.59556680	-1.79857228	0.97895235
H	5.41208914	7.86059973	-0.21388466
H	7.90153165	7.92254739	-0.24980089
H	9.19118232	5.78527510	-0.23914135
H	8.01578440	3.63599211	-0.19326274
H	-9.51923036	0.75543551	0.08003507
H	-10.81938076	2.87996656	0.05094256
H	-9.61387921	5.06524507	-0.00486279
H	-7.16484632	5.12233932	-0.03178537
H	9.42615078	-3.91343307	0.94866094
H	9.11688594	-4.11477883	-0.77330624
H	10.95517484	-1.21022518	-0.20855531

H	9.62311780	-0.11549572	-0.39089421
H	-8.08645941	-5.83049176	0.93016647
H	-8.14916284	-6.22091376	-0.78637248
H	-4.90508960	-8.29372766	0.36025517
H	-6.53001730	-8.88798662	0.25214354
H	-0.94403933	10.05260001	-0.32291520
H	-2.39949435	10.98712439	-0.20541703
H	-4.58959976	8.34315673	-0.88340970
H	-4.55469911	8.72996710	0.83440488
H	11.39576247	-4.67388224	-0.33938459
H	11.39473513	-3.19484731	-1.31200042
H	11.75841769	-3.12749204	0.42817324
H	10.69350224	0.19503180	1.83703841
H	9.04739444	-0.42244976	2.07667033
H	10.43571831	-1.51355277	2.23650235
H	-5.30028976	-9.31078501	-1.87851494
H	-6.66415495	-8.21606561	-2.17263951
H	-5.01438512	-7.57160223	-2.08285795
H	-9.73121670	-7.52473109	0.60808297
H	-8.62287723	-8.62236562	-0.21522694
H	-8.39784297	-8.25999912	1.51189401
H	-5.82665565	10.35412680	-0.54086807
H	-4.50012362	11.18000145	0.27722625
H	-4.37262867	10.78869425	-1.45311740
H	-1.10622774	11.12146212	1.92256131
H	-2.67054585	10.33932285	2.21692985
H	-1.19546952	9.35992155	2.11520040

Table S9. Optimized Cartesian coordinates (Å) of CQDs-NPr_2 for the first excited state.

C	3.73302500	0.26988700	-0.01653000
C	2.59777800	1.11192000	-0.04264200
C	1.29346900	0.55237900	-0.06218100
C	1.12522800	-0.85283700	-0.05589000
C	2.27328700	-1.69194800	-0.02918600
C	3.57321800	-1.13802900	-0.01364900
C	0.15115800	1.41359800	-0.09120600
C	-0.19152800	-1.41144000	-0.06912700
C	0.30319600	2.82770900	-0.09873000
C	-0.82794900	3.67483000	-0.13253700
C	-2.12739600	3.10924700	-0.15338500
C	-2.28855400	1.70434300	-0.13551800
C	-1.15060600	0.85656400	-0.10544100
C	-1.32481300	-0.56336200	-0.09390800
C	-2.62531200	-1.13902300	-0.10275700

C	-2.79448200	-2.54149800	-0.09399600
C	-1.65485000	-3.38334800	-0.07166000
C	-0.35788300	-2.82093700	-0.06066600
N	-3.25250800	3.92324200	-0.18572300
C	4.72204300	-2.01230400	0.01792500
C	2.10779700	-3.12102200	-0.02101200
C	0.79988400	-3.66781600	-0.03765400
C	0.61587800	-5.06684500	-0.03352000
C	1.72853100	-5.94453300	-0.01900700
C	3.01349600	-5.38186000	-0.00504500
C	3.23320800	-4.00132200	0.00127800
C	4.58457100	-3.42412000	0.02637600
C	5.73498100	-4.22885700	0.07558500
C	7.03243300	-3.68702900	0.08936100
C	7.13685200	-2.27172200	0.08012600
C	6.01616700	-1.46229900	0.04884800
C	1.62480600	3.39627700	-0.07006600
C	-0.64593700	5.11131900	-0.13055300
C	-3.78114400	-0.28210700	-0.12550100
C	-4.12561200	-3.10114600	-0.09835500
C	-3.60094800	1.12380100	-0.14472200
C	-4.72121100	1.98132100	-0.17566700
C	-6.03721100	1.45597000	-0.19082700
C	-6.19185500	0.06177400	-0.16854500
C	-5.10613000	-0.81754500	-0.13159200
C	-5.28177500	-2.27733700	-0.10538800
C	-6.55132500	-2.87173300	-0.08565900
C	-6.73613700	-4.26912800	-0.08344200
C	-5.55993700	-5.06442400	-0.06532800
C	-4.29801300	-4.49429800	-0.07850800
C	0.65119100	5.68889800	-0.08733500
C	0.76530800	7.09109400	-0.08793200
C	-0.35111500	7.92329300	-0.10629200
C	-1.62736500	7.33124200	-0.14518800
C	-1.77338800	5.95123900	-0.16973700
C	2.75216300	2.53739600	-0.04293300
C	4.05508900	3.07909000	-0.01508600
C	4.25879300	4.48166800	-0.00950400
C	3.12895600	5.31311100	-0.03057500
C	1.82548000	4.80988100	-0.06265900
C	-3.16875500	5.39195300	-0.24600600
C	-4.54597000	3.40734700	-0.20307200
N	-5.53578000	4.27036700	-0.24848000
C	-6.82590500	3.78706300	-0.26080300
C	-7.87501900	4.73494800	-0.30477800
C	-9.19809900	4.33188600	-0.31826500
C	-9.51648600	2.96115600	-0.28790300

C	-8.50428700	2.01591900	-0.24518600
C	-7.14531100	2.39879200	-0.23154600
N	-1.80029800	-4.76517200	-0.06028700
N	5.00209200	0.83592400	0.00722000
C	6.23346100	0.02804000	0.02943400
C	5.20265000	2.21356900	0.00880800
C	-0.70760000	-5.62799000	-0.04599700
C	-3.11586600	-5.42764300	-0.06499200
N	-0.96055200	-6.91770100	-0.04391400
C	6.67221800	3.99874600	0.03665100
C	8.01802400	4.43414800	0.06174300
C	8.32988400	5.78172900	0.06748100
C	7.30158100	6.74276200	0.04833400
C	5.97666000	6.33831600	0.02404800
C	5.62953100	4.96965600	0.01765900
N	6.44570200	2.63986500	0.03187800
C	0.10296300	-7.79343600	-0.03069600
C	-0.19360800	-9.17668400	-0.02927100
C	0.81671800	-10.12133700	-0.01594800
C	2.16319900	-9.71177800	-0.00325800
C	2.47602500	-8.36191100	-0.00422700
C	1.46518000	-7.37619700	-0.01820600
N	8.17981400	-4.47266600	0.10170300
N	-0.16292300	9.34317800	-0.08901600
N	-7.99947100	-4.84675300	-0.11831900
C	-1.07670100	10.09437400	-0.97080800
C	-0.10866300	9.91307500	1.27567200
C	-8.09625700	-6.31171200	-0.00541300
C	-9.17695500	-4.03714100	0.20674900
C	9.40002100	-3.94451500	0.73075600
C	8.07565900	-5.93020500	-0.01208300
C	-0.42594000	11.36043000	-1.54228900
C	1.15254100	9.56076900	2.06834500
C	-9.36326900	-3.74645500	1.70552900
C	-9.48288100	-6.90814700	-0.26527700
C	9.27973400	-6.59961200	-0.68852200
C	10.44815000	-3.38372300	-0.24424600
C	1.17849800	10.25518800	3.43478200
C	-1.37459800	12.12944300	-2.46809800
C	-10.60498600	-2.89148400	1.97642300
C	-9.42030300	-8.44046300	-0.27621800
C	11.67669600	-2.84029700	0.49259500
C	9.06845400	-8.11180600	-0.82456700
H	3.86274200	-6.05056800	-0.00562800
H	5.61662000	-5.29943300	0.14241400
H	8.11354500	-1.80063900	0.07293300
H	-7.19511400	-0.33991200	-0.19001300

H	-7.41981100	-2.23216500	-0.06926800
H	-5.62601700	-6.14456100	-0.02818600
H	1.73621500	7.56978900	-0.09480000
H	-2.51368200	7.96002100	-0.15860800
H	3.27923700	6.38355400	-0.02093100
H	-3.65952800	5.71720900	-1.17183700
H	-3.78662000	5.79297400	0.56446200
H	-7.60530900	5.78675300	-0.32666600
H	-9.99173200	5.07334400	-0.35166100
H	-10.55465300	2.64199100	-0.29748700
H	-8.77132000	0.96415700	-0.22080800
H	6.83571200	0.31505300	-0.84070400
H	6.81932300	0.33981100	0.90155000
H	-3.15891900	-6.08721900	0.80958600
H	-3.14738000	-6.09659900	-0.93289300
H	8.79480000	3.67527000	0.07615600
H	9.36908100	6.09874900	0.08658800
H	7.54430000	7.80143600	0.05254100
H	5.19835900	7.09462200	0.00911300
H	-1.23943200	-9.46946200	-0.03890900
H	0.57083400	-11.17979500	-0.01504800
H	2.95838900	-10.45156400	0.00763200
H	3.52045900	-8.06680600	0.00700600
H	-1.35041700	9.43994800	-1.80528000
H	-2.01737700	10.36187100	-0.45617800
H	-0.15303200	11.00163000	1.16676900
H	-1.00397200	9.61910200	1.85473400
H	-7.41596900	-6.74657600	-0.74621300
H	-7.74150200	-6.65017400	0.98276600
H	-9.12980700	-3.09911400	-0.35559000
H	-10.05838500	-4.55379500	-0.17662700
H	9.11945300	-3.16856900	1.45273600
H	9.85167100	-4.75166900	1.32051600
H	7.91007200	-6.39088800	0.97762700
H	7.19843300	-6.15927700	-0.62130800
H	-0.10121100	12.02014000	-0.72753300
H	0.48118400	11.06833500	-2.08613700
H	1.21564200	8.47559000	2.21092200
H	2.03249700	9.85285100	1.48058600
H	-9.44086000	-4.69871300	2.24682100
H	-8.46916500	-3.24106300	2.09127500
H	-10.19596000	-6.58716800	0.50289200
H	-9.86458000	-6.54972800	-1.22968900
H	9.42576000	-6.15118900	-1.67913500
H	10.19725400	-6.41572600	-0.11785000
H	10.75005500	-4.17074900	-0.94470100
H	9.99573600	-2.58993200	-0.85172700

H	2.08372700	9.99125500	3.99245500
H	1.15813200	11.34674500	3.32815300
H	0.31471400	9.96474300	4.04532900
H	-0.89052100	13.02525900	-2.87219200
H	-1.69216600	11.51014600	-3.31609100
H	-2.27772000	12.45030900	-1.93462400
H	-10.72407000	-2.69606100	3.04766300
H	-10.53932300	-1.92267100	1.46583400
H	-11.51622600	-3.39090600	1.62489900
H	-10.41441400	-8.86744000	-0.44499000
H	-8.75805800	-8.80694500	-1.06948700
H	-9.04687400	-8.82983200	0.67846700
H	12.41655800	-2.44568900	-0.21206700
H	11.40279800	-2.02939000	1.17878400
H	12.16433000	-3.62511800	1.08405200
H	9.93247700	-8.58516600	-1.30285200
H	8.92939800	-8.58317300	0.15591100
H	8.18375300	-8.33769800	-1.43204100

Table S10. The calculated absorption wavelength (λ_{Abs}, nm), absorption oscillator strength (f_{Abs}), PL emission wavelength (λ_{Em}, nm), emission oscillator strength (f_{Em}), HOMO (eV), LUMO (eV) and bandgap energies (eV) of CQDs-0, –NH_2, –NMe_2, –NEt_2, and –NPr_2.

	CQDs-0	CQDs-NH_2	CQDs-NMe_2	CQDs-NEt_2	CQDs-NPr_2
λ_{Abs}	482	498	518	530	534
f_{Abs}	0.110	0.272	0.827	0.858	0.869
λ_{Em}	559	583	614	620	623
f_{Em}	0.245	0.446	1.165	1.262	1.271
HOMO	-5.30	-5.15	-5.02	-4.95	-4.90
LUMO	-2.42	-2.38	-2.35	-2.31	-2.30
GAP	2.88	2.77	2.67	2.64	2.60

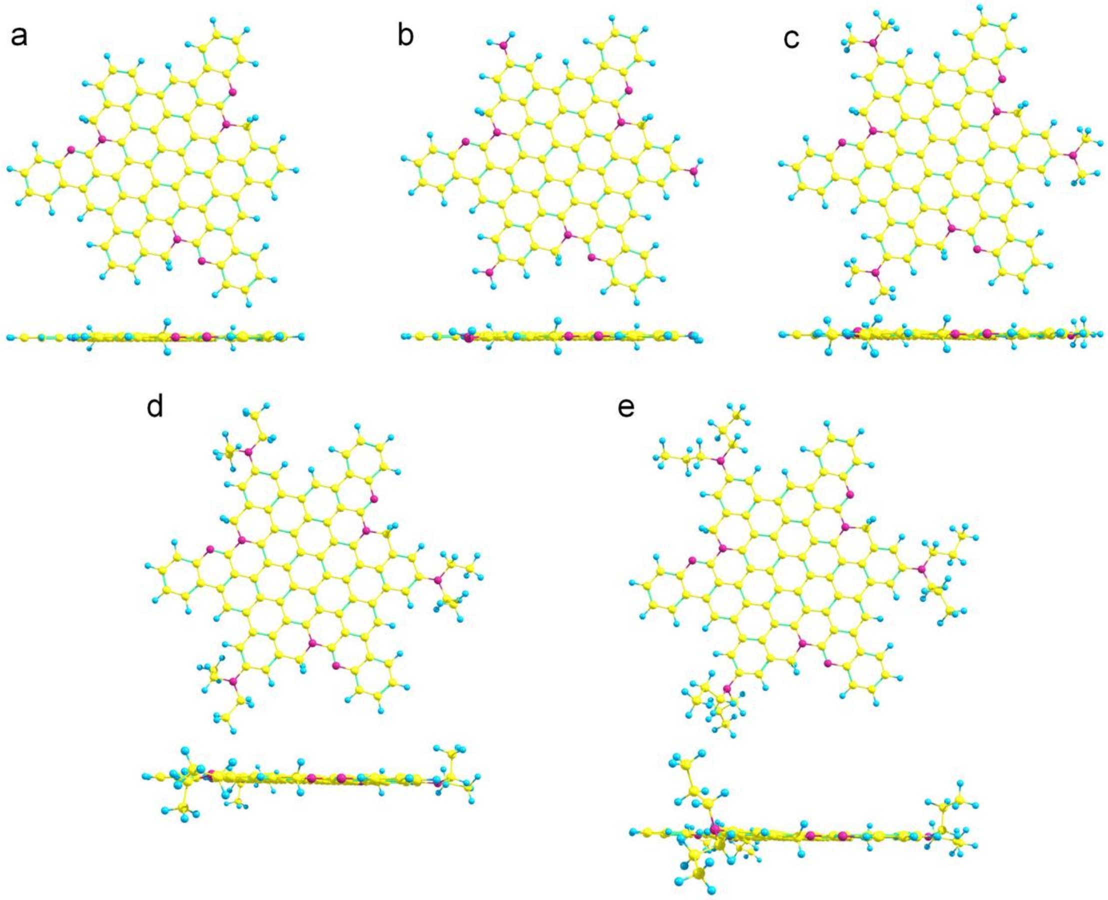

Figure S9. Optimized structures of CQDs-0 (a),- NH_2 (b), -NMe_2 (c), -NEt_2 (d), and -NPr_2 (e). Top view (top), side view (bottom).

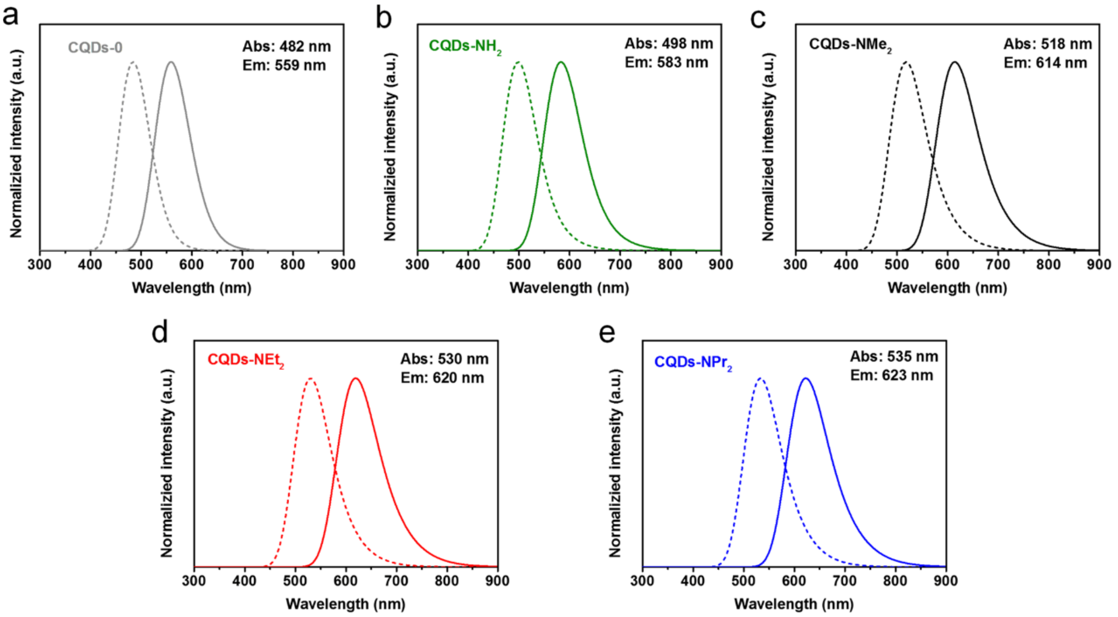

Figure S10. The calculated UV-vis absorption (dashed line) and PL emission spectra (solid line) of CQDs-0 (a),-NH_2 (b), -NMe_2 (c), -NEt_2 (d), and -NPr_2 (e).

Control experiment:

In order to further affirm the effect of passivating electron-donating group (EDG) on the optical properties of CQDs, other two CQDs (namely CQDs-OMe and -Mor) passivated by relatively weak EDG, e.g., methoxy group (-OMe) and morpholinyl group (-Mor) have been synthesized by selecting 4-methoxy-aniline and 4-morpholinyl-aniline as precursors. The synthesis and purification process remain the same as those of R-EPG-CQDs-NMe_2, -NEt_2, and -NPr_2. In comparison with R-EPG-CQDs-NMe_2, -NEt_2, and -NPr_2, CQDs-OMe and -Mor exhibit lower QY (10 and 34%) and shorter PL emission (597 and 631 nm) in dilute ethanol solution (Figure S11). As shown in the statistical comparison of the optical properties among these CQDs (Figure S12), increasing the electron-donating ability of passivating group on CQDs is beneficial to acquire higher QY and longer PL emission, which agrees well with the theoretical investigation.

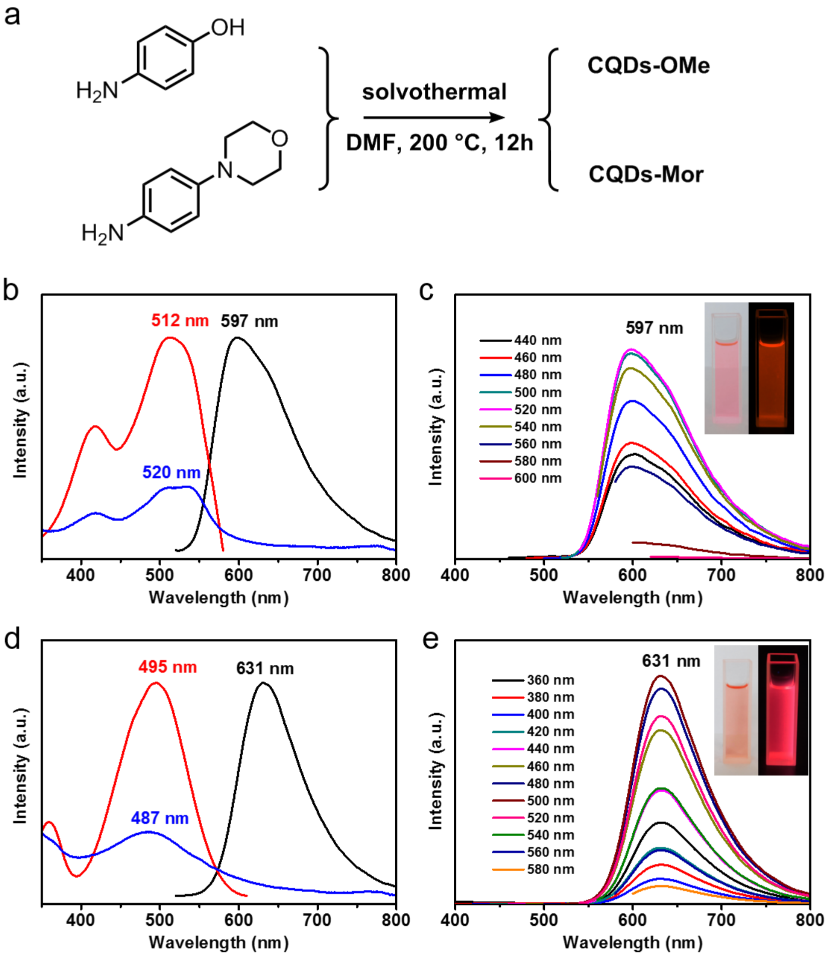

Figure S11. The synthesis (a) of CQDs-OMe and –Mor. UV-vis absorption (blue line), normalized PL emission (black line, fixed excitation wavelength at 500 nm) and excitation (red line, fixed emission wavelength at 600 and 630 nm, respectively) spectra of CQDs-OMe (b) and –Mor (d) dilute ethanol solution. PL emission spectra under different excitation wavelength and photographs under daylight (left inset) and 365 nm UV light (right inset) of CQDs-OMe (c) and –Mor (e) dilute ethanol solution.

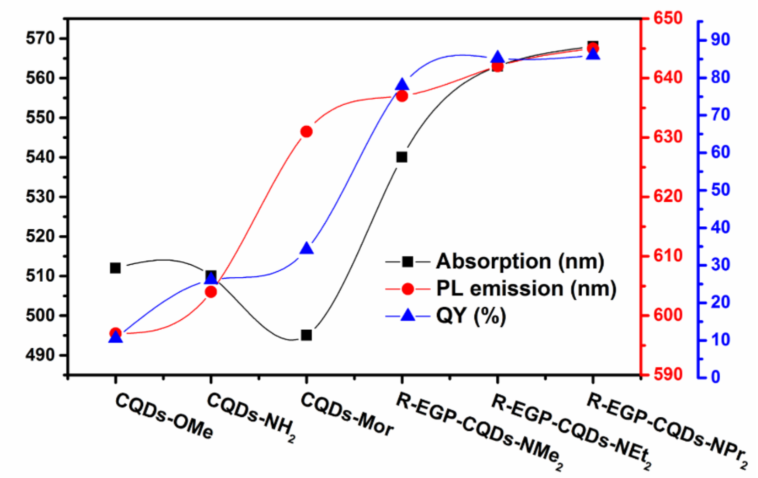

Figure S12. The statistical comparison of the optical properties among CQDs-OMe, CQDs-NH_2 (Ref. 24), CQDs-Mor, R-EGP-CQDs-NMe_2, -NEt_2, and -NPr_2.

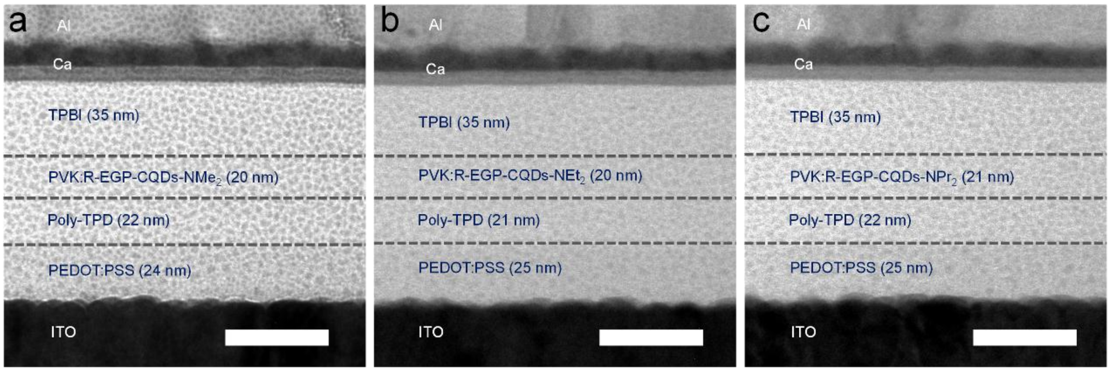

Figure S13. The cross-sectional TEM images of WLEDs-1 (a), -2 (b) and -3 (c) based on the ITO/PEDOT:PSS/Poly-TPD/PVK:R-EGP-CQDs (9 wt%)/TPBI /Ca/Al structure. Scale bar: 50 nm.

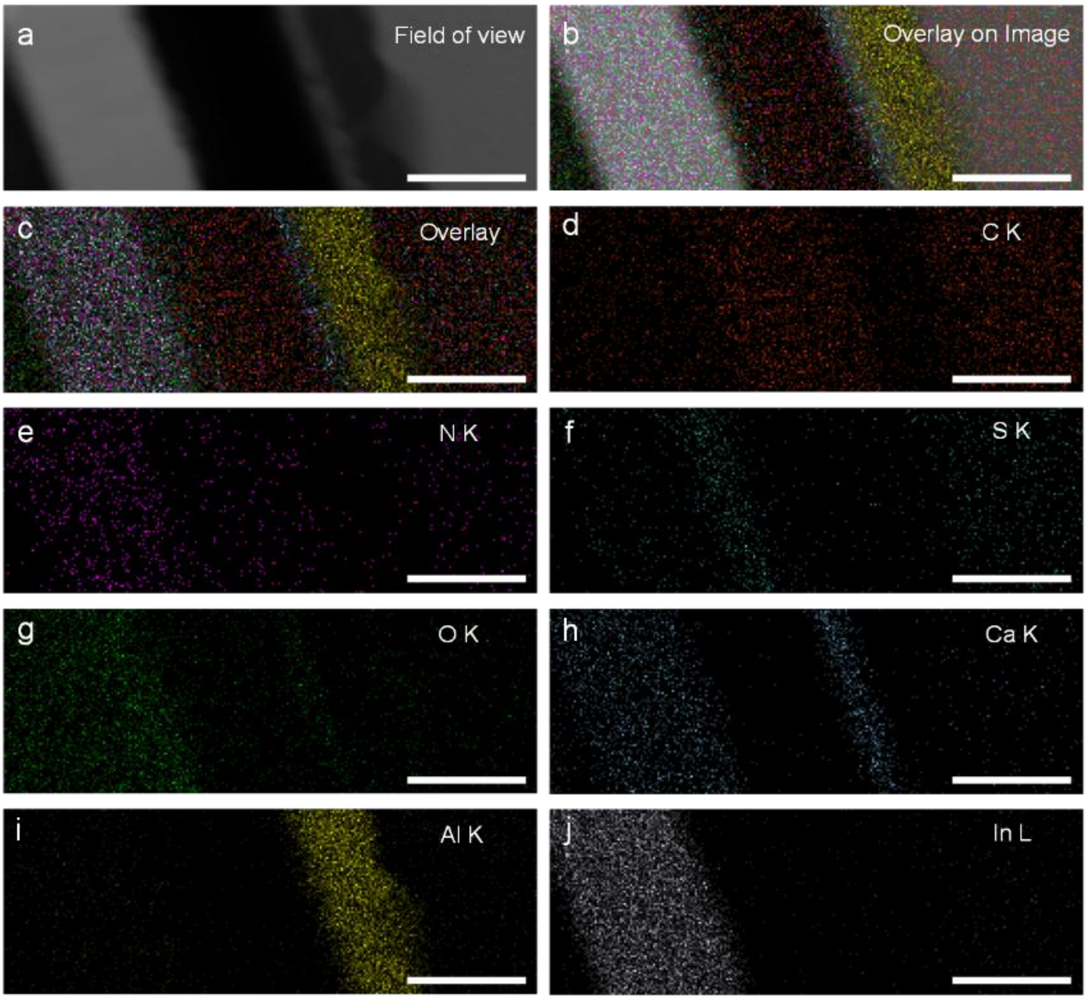

Figure S14. The cross-sectional TEM image (a) and corresponding EDX mapping images (b-j) of a typical WLEDs device. Scale bar: 100 nm.

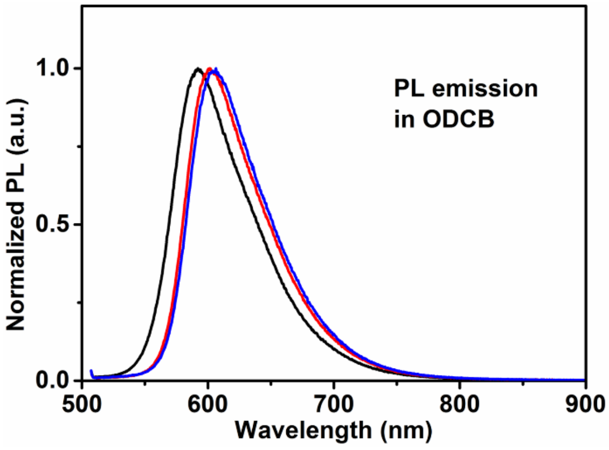

Figure S15. Normalized PL emission spectra in ODCB of R-EGP-CQDs-NMe_2 (black), -NEt_2 (red), and –NPr_2 (blue) with 500 nm excitation.

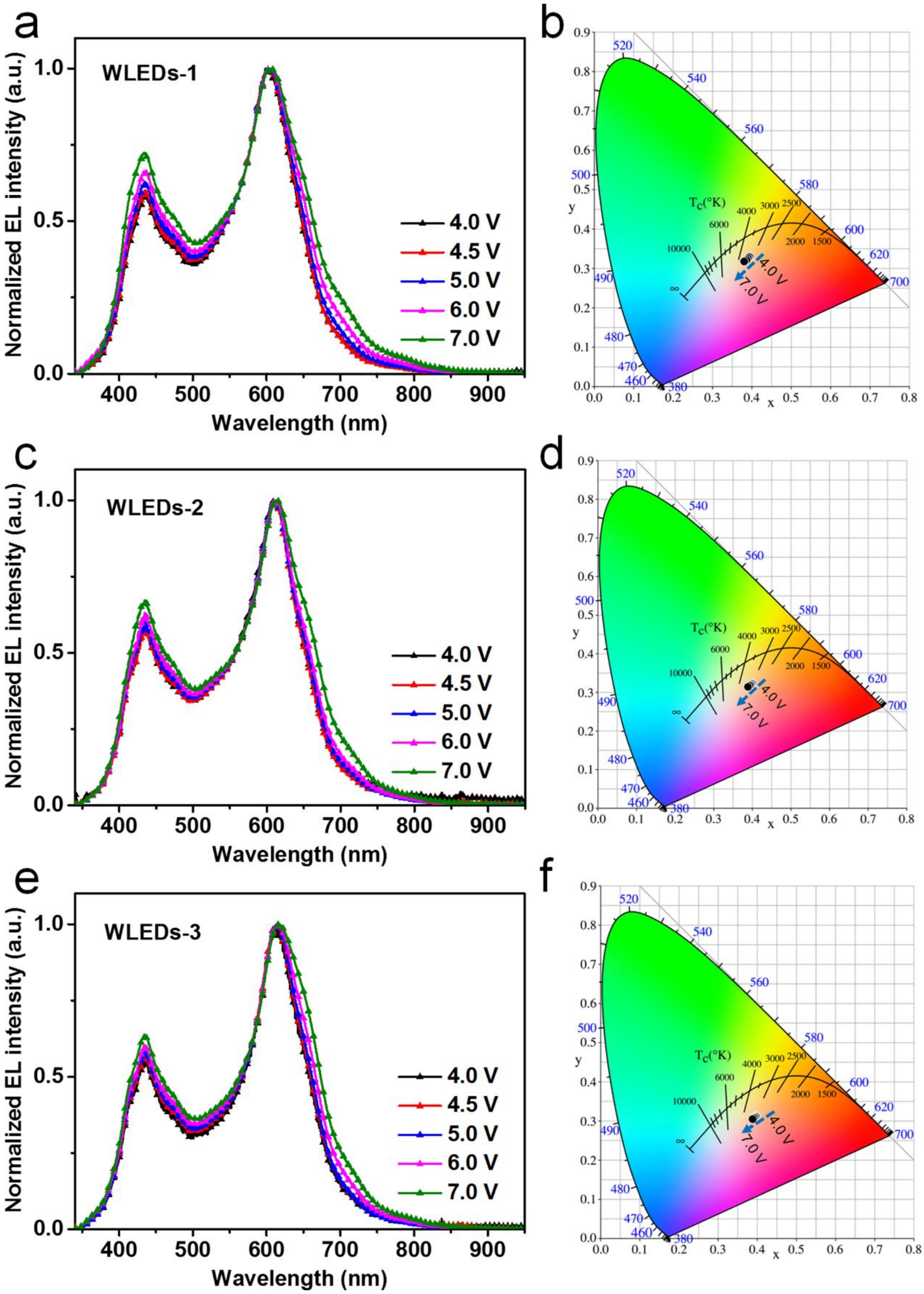

Figure S16. Normalized EL spectra (a, c, e), and corresponding CIE coordinates (b, d, f) of the optimized WLEDs-1, -2, and -3 at different bias voltages.

Table S11. Detailed device performances of optimized WLEDs-1, -2, and -3 at different bias voltage.

LEDs	EML	Bias Voltage [V]	Luminance [cd/m^2]	Current Density [mA/cm^2]	Current Efficiency [cd/A]	EL peak [nm]	CIE		CCT [K]
							x	y	
WLEDs-1	PVK: R-EGP-CQDs-NMe_2 (9 wt%)	4.0	98	7	1.40	602/435	0.398	0.330	2984
		4.5	371	12	2.96	603/435	0.396	0.328	3031
		5.0	933	26	3.49	604/435	0.392	0.325	3087
		6.0	2956	96	3.06	605/435	0.388	0.321	3170
		7.0	5004	251	1.98	605/434	0.379	0.314	3365
WLEDs-2	PVK: R-EGP-CQDs-NEt_2 (9 wt%)	4.0	148	5	2.73	610/435	0.398	0.324	2894
		4.5	451	13	3.41	611/435	0.397	0.323	2920
		5.0	1083	29	3.70	611/435	0.394	0.320	2977
		6.0	3334	111	2.99	612/435	0.390	0.317	3048
		7.0	5765	314	1.84	612/435	0.383	0.311	3168
WLEDs-3	PVK: R-EGP-CQDs-NPr_2 (9 wt%)	4.0	200	6.6	3.03	613/435	0.401	0.315	2665
		4.5	601	16	3.71	613/435	0.399	0.315	2725
		5.0	1339	36	3.71	614/435	0.396	0.313	2789
		6.0	3628	132	2.74	615/435	0.393	0.313	2868
		7.0	5875	344	1.70	616/435	0.388	0.309	2987

Table S12. Comparison of our CQDs-based warm-WLEDs with other previously reported representative CQDs-based and Colloidal semiconductor QDs-based WLEDs.

WLEDs		V_{on} [V]	L_{max} [cd/m^2]	$\eta_{c,max}$ [cd/A]	CIE	CCT [K]	Reference
Carbon quantum dots (CQDs)	PVK: R-EGP-CQDs -NMe_2/-NEt_2/-NPr_2	3~3.3	5248~5909	3.65~3.85	(0.383,0.311)	2987~3365	This work
	PVK:G-CQDs	3.9	2050	1.1	(0.30, 0.33)	7227	Adv. Mater. 2017, 29, 1604436.
	PVK: CDs	~8	455.2	-	(0.29, 0.33)	7694	Nanoscale, 2018, 10, 11211
	WCQDs	5	300	-	(0.27, 0.29)	11010	Adv. Funct. Mater. 2016, 26, 2739-2744.
	CDs	4.6	90	-	-	-	ACS nano 2013, 7, 11234-11241.
	CQDs	5.7	35	0.022	-	-	Chem. Commun. 2011, 47, 3502-3504.
Colloidal semiconductor	mixed RGB Cd^{2+}-QD active	~5	23352	21.8	(0.319, 0.336)	6126~7719	ACS Nano 2015, 9, 10941-10949.

quantum dots (QDs)	layers						
	mixed Cd^{2+}-QD active layers	~4	6400	-	(0.35, 0.39)	3900~8000	Adv. Mater. 2014, 26, 6387-6393.
	CBP:QDs (B, G, R)	6	1050	1.8	(0.32,0.45)	5897	Adv. Mater. 2006, 18, 2545-2548.
	CGS/ZnS QDs	5~6	924	2.9	(0.332,0.338)	4700	Adv. Mater. 2016, 28, 5093-5098.
	RBG-QDs	~5	830	0.9	(0.35, 0.41)	5500	Nano Lett. 2007, 7, 2196-2200.
	ZnCuInS/ZnS QDs/poly-TPD	10	450	-	(0.336,0.339)	5310	Nano Lett. 2011, 11, 329-332.
	InP/ZnS /poly-TPD	11	270	-	(0.332,0.338)	5000~6000	Adv. Mater. 2012, 24, 4180-4185.
	TPD/CdSe	-	4.83×10^{-5} lumen/watt	-	(0.333,0.333)	5500~6000	Nano Lett. 2010, 10, 573-576.

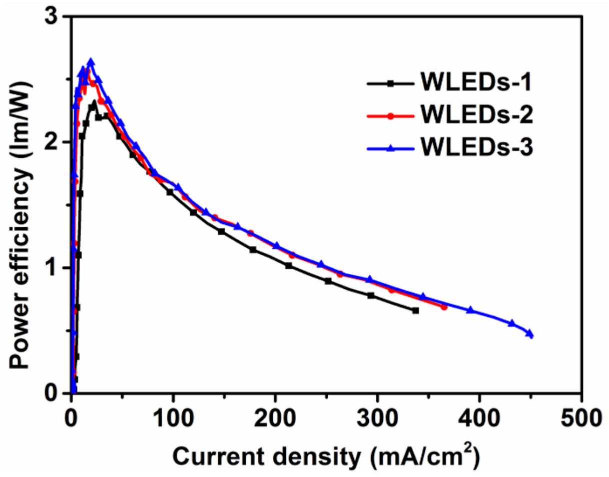

Figure S17. The power efficiency curves of WLEDs-1, -2, and -3.

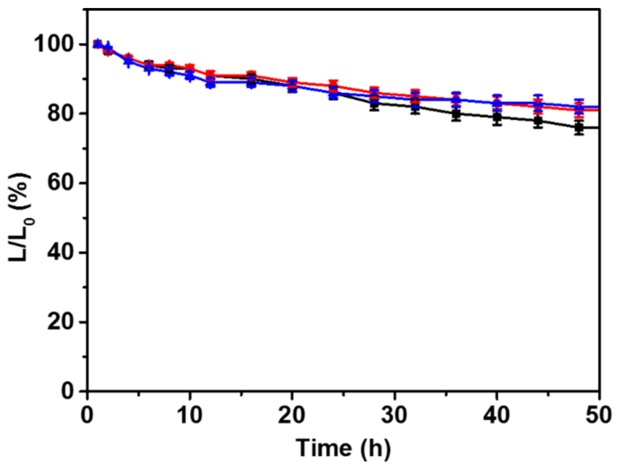

Figure S18. The operation stability plots of WLEDs-1 (black), -2 (red), and -3 (blue).

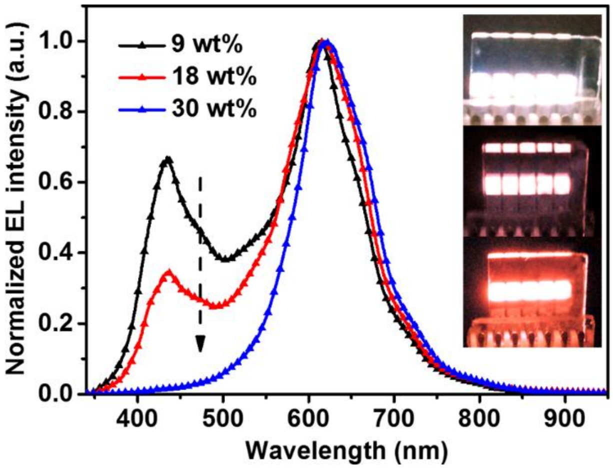

Figure S19. EL spectra and operation photographs (from top to bottom) of LEDs at 7 V with 9, 18, and 30 wt% R-EGP-CQDs-NEt_2 in PVK as EML, respectively.

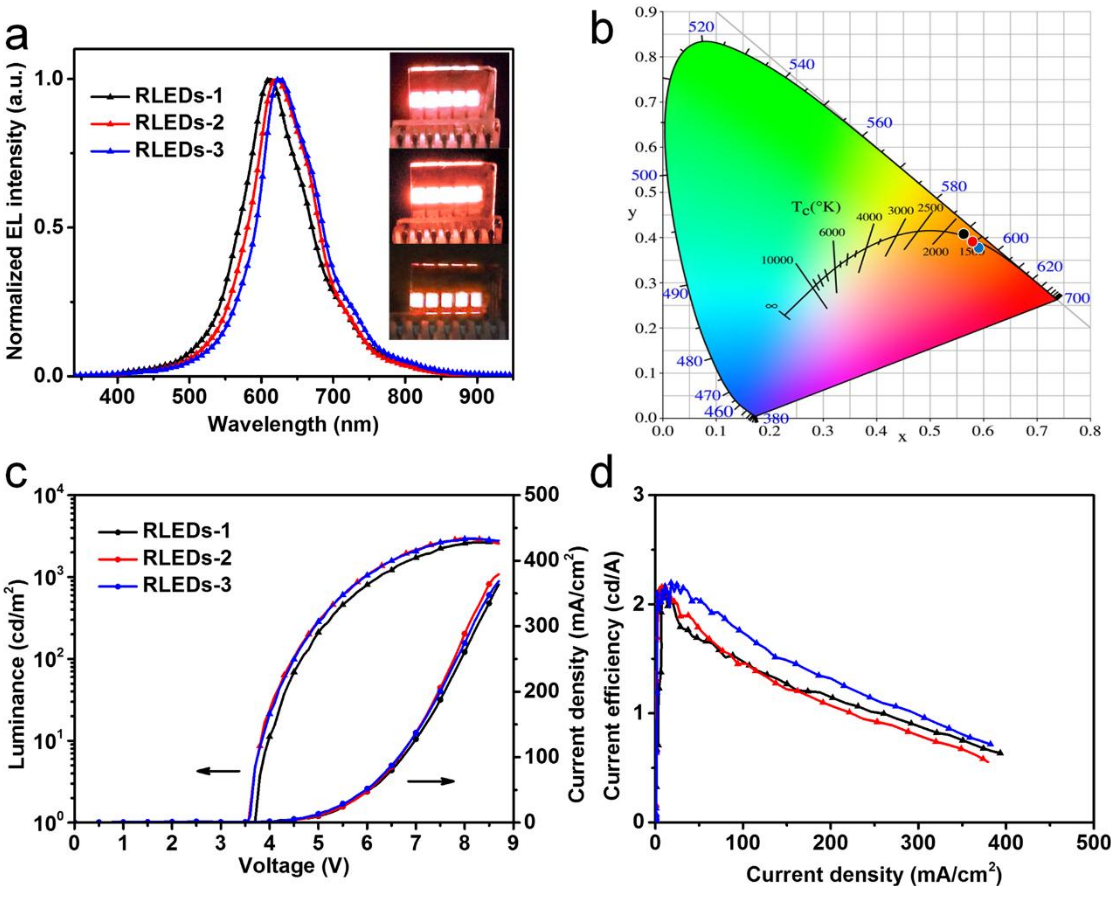

Figure S20. EL spectra (a), operation photographs (insets in a, from top to bottom), and corresponding CIE coordinates (b) of RLEDs-1, -2, and -3 under 7.0 V, respectively. The J-V-L (c) and current efficiency (d) of RLEDs-1 (black), -2 (red), and -3 (blue).

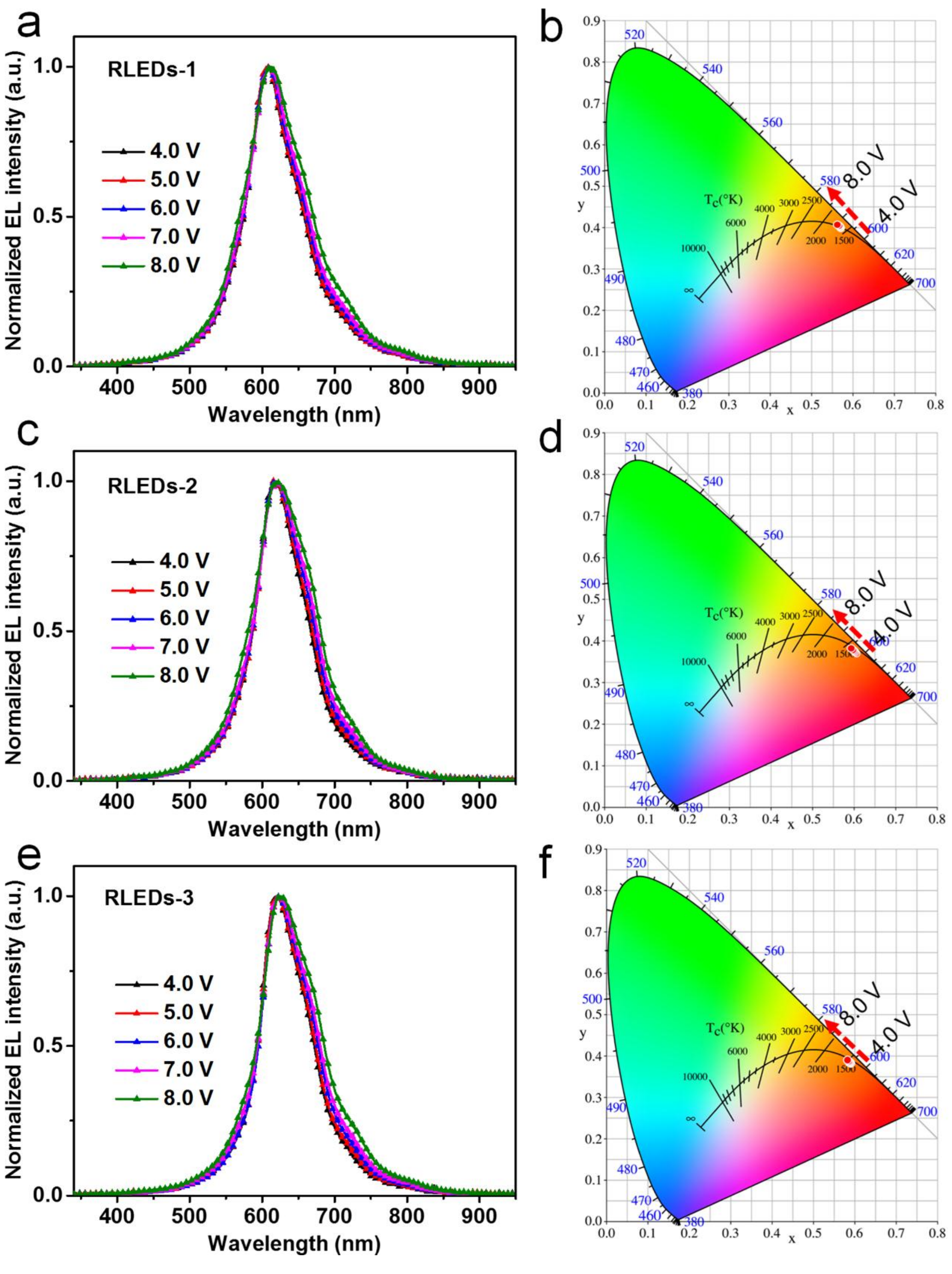

Figure S21. Normalized EL spectra (a, c, e), and corresponding CIE coordinates (b, d, f) of the optimized RLEDs-1, -2, and -3 at different bias voltages.

Table S13. Device performances of optimized RLEDs-1, -2, and -3 at different bias voltage.

LEDs	EL peak [nm]	V_{on} [V]	L_{max} [cd/m^2]	$\eta_{c,\ max}$ [cd/A]	CIE [e]
RLEDs-1	611	3.7	2687	2.11	(0.564, 0.404)
RLEDs-2	621	3.6	2933	2.17	(0.581, 0.390)
RLEDs-3	626	3.6	2960	2.19	(0.591, 0.378)
